# Allelic variation and genetic diversity of HMW glutenin subunits in Chinese wheat (*Triticum aestivum* L.) landraces and commercial cultivars

**DOI:** 10.1270/jsbbs.21076

**Published:** 2022-02-02

**Authors:** Xiaofang Wang, Ruilian Song, Yue An, Haiyi Pei, Song Gao, Daokun Sun, Xifeng Ren

**Affiliations:** 1 College of Plant Science and Technology, Huazhong Agricultural University, Wuhan, 430070, China; 2 Hubei Hongshan Laboratory, Wuhan, 430070, China

**Keywords:** wheat landraces, wheat commercial varieties, HMW-GS, SDS-PAGE, genetic diversity

## Abstract

Wheat landraces have abundant genetic variation at the *Glu-1* loci, which is desirable germplasms for genetic enhancement of modern wheat varieties, especially for quality improvement. In the current study, we analyzed the allelic variations of the *Glu-1* loci of 597 landraces and 926 commercial wheat varieties from the four major wheat-growing regions in China using SDS-PAGE. As results, alleles Null, 7+8, and 2+12 were the dominant HMW-GSs in wheat landraces. Compared to landraces, the commercial varieties contain higher frequencies of high-quality alleles, including 1, 7+9, 14+15 and 5+10. The genetic diversity of the four commercial wheat populations (alleles per locus (A) = 7.33, percent polymorphic loci (P) = 1.00, effective number of alleles per locus (Ae) = 2.347 and expected heterozygosity (He) = 0.563) was significantly higher than that of the landraces population, with the highest genetic diversity found in the Southwestern Winter Wheat Region population. The genetic diversity of HMW-GS is mainly present within the landraces and commercial wheat populations instead of between populations. The landraces were rich in rare subunits or alleles may provide germplasm resources for improving the quality of modern wheat.

## Introduction

Wheat (*Triticum aestivum* L.) is one of the three main cereal crops in the world. It is a staple food and an important source of protein for humans (Payne 1987). The gluten protein in wheat grain is the chemical bases for making various end-products. Its processing quality is mainly determined by seed storage proteins that consist of polymeric glutenins and monomeric gliadins (Ma *et al.* 2019, Shewry *et al.* 1986). Glutenin plays a major role in dough elasticity, while gliadin mainly affects dough viscosity (Anjum *et al.* 2007, Ciaffi *et al.* 1996). According to the relative mobility of the glutenins in sodium dodecyl sulfate-polyacrylamide gel electrophoresis (SDS-PAGE), glutenin proteins are divided into two groups: high-molecular-weight glutenin subunits (HMW-GS) and low-molecular-weight glutenin subunits (LMW-GS) (Payne 1987). Although HMW-GS only accounts for 10% of the total gluten proteins, it is the main component of gluten polymer, as the “network backbone” of gluten protein, and plays a decisive role in gluten elasticity (Don *et al.* 2006, Ma *et al.* 2019, Payne *et al.* 1980, Shewry *et al.* 1992).

HMW-GS are encoded by a multi-gene family located at *Glu-A1*, *Glu-B1* and *Glu-D1* loci on the long arms of chromosomes 1A, 1B and 1D, respectively (Payne *et al.* 1987). Previous studies confirmed each locus contains two tightly-linked genes encoding the x- and y-type subunits: the larger x-type subunits with 80–88 kDa and the smaller y-type subunits with 67–73 kDa (Harberd *et al.* 1986, Liu *et al.* 2009, Shewry *et al.* 2003). Compared with y-type HMW-GS, the x-type HMW-GS has a greater contribution to gluten strength (Zhang *et al.* 2007). Most common bread wheat cultivars usually express only three to five subunits because some genes encoding the y subunits are silent (Roy *et al.* 2021, Shewry *et al.* 2003). Therefore, in common bread wheat, 1Ax and 1Ay subunits are encoded by *Glu-A1*, 1Bx and 1By encoded by *Glu-B1*, and 1Dx and 1Dy encoded by *Glu-D1* (Payne 1987, Payne and Lawrence 1983, Yu *et al.* 2019). Various studies have shown that the *Glu-1* locus exhibits different allelic variations and gene inactivation in bread wheat, which are closely related to the end-use quality (Ma *et al.* 2005, Shewry *et al.* 1992). The quality and quantity of HMW-GS has a profound influence on the quality of bread-making and dough properties of wheat flours (Payne 1987).

The allelic composition and expression level of HMW-GS are strongly associated with dough properties and bread-making quality (Gupta *et al.* 1993, Payne 1987, Roy *et al.* 2018, 2020, Shewry *et al.* 1992). Particularly, some subunits such as 1Dx5+1Dy10, 1Bx14+1By15, and 1Ax1 have been associated with superior bread-making quality while 1Dx2+1Dy12, 1Bx20 have negative effects on dough strength (Altpeter *et al.* 1996, Redaelli *et al.* 1997). Subunits 1Ax2* and 1Dx17+1Dy18 also have a positive impact on bread-making quality (Ma *et al.* 2005, Payne *et al.* 1987). The high expression levels of some subunits also show positive effects on wheat processing quality (Butow *et al.* 2003). In particular, two functional copies of the 1Bx7^OE^ subunit significantly improve the dough strength (Ragupathy *et al.* 2008). The low frequency of subunit 5+10 and high frequency of subunit 2+12 are partially responsible for the typical weak gluten characteristics of Chinese wheat commercial varieties (He *et al.* 1992). In recent years, the Chinese wheat breeding priority has been shifted from yield to quality, resulted in an increased demand for germplasms with a broad diversity of HMW-GS alleles (Hu and Wang 2016). A trend occurred in recent years marks the exploration of the glutenin diversity in wheat relatives (Zhang *et al.* 2018). Nevertheless, the glutenin diversity in the vast number of Chinese wheat landraces characterized by heterogeneity and rich genetic backgrounds has not been systematically studied (Newton *et al.* 2010).

The specific distribution of subunit combinations in the main Chinese wheat growing areas is not clear. This study focuses on the distribution of subunit or allele combinations in the main wheat growing regions of China. As results, a large number of new subunit combinations were only found in landraces. Narrow genetic diversity was found for the subunit combinations of the Chinese wheat commercial varieties, especially in the southwest winter wheat region. These results are potentially useful for Chinese wheat breeding.

## Materials and Methods

### Plant materials

A total of 1523 Chinese common bread wheat lines were analyzed (Supplemental Table 1), including 597 landraces and 926 commercial varieties. The 597 landraces are from Huang-huai Winter Wheat Region (HWWR) and Middle and Low Yangtze Winter Wheat Region (MLYWWR). The commercial varieties are from four major wheat growing areas in China, including 504 from HWWR, 131 from MLYWWR, 133 from Southwestern Winter Wheat Region (SWWR), 158 from Northwest Spring Wheat Region (NSWR). Among them, the 635 wheat varieties from HWWR and MLYWWR were used for comparative analysis with wheat landraces. Since the HWWR is the main wheat growing area in China, the number of commercial varieties collected from this region was rather large. Due to the small number of NSWR region commercial varieties held in our laboratory, data of 71 NSWR commercial varieties were cited from another study (Zheng *et al.* 2020). Cultivars Zhongyou9507 and Chinese spring were used as standard cultivars for allele identification.

### Electrophoretic analysis

For the protein extraction, three individual seeds were ground to a fine powder using a tissue grinder (TissueLyser II). Meal flours of 40 mg were extracted into 600 μl SDS-PAGE sample buffer (62.5 mM Tris-HCl pH 6.8, 2% (w/v) SDS, 10% (v/v) glycerol, 5% (v/v) 2-mercaptoethanol, 0.002% (w/v) bromophenol blue), 2 min under continuous vortex mixing, and shaken for 30 min, placed in a 90°C boiling water bath for 5 min. The mixtures were immediately centrifuged for 5 min at 13,000 rpm, and 8 μl of supernatant was used for SDS-PAGE analysis. The SDS-PAGE was conducted with 12% (w/v) running gels and 4% (w/v) stacking gels. In an electrode buffer of 1 times Tris-glycine, the gels were carried out at a constant current of 25 mA for about 5 h (Liuyi-Beijing mini-cell apparatus). After electrophoresis, the gel plate was placed in 0.05% (w/v) Coomassie Brilliant blue solution (R-250) for 8–10 h, and then decolorized with water for 10–12 h. HMW-GS was classified using the nomenclature of Payne and Lawrence (1983).

### Statistical methods

Microsoft Excel was used to calculate number of allele combinations and the allelic frequencies. The method of Gao *et al.* (2020) was used for cluster analysis with some modifications. Subunit band was recorded as “0 and 1”, with “1” as present and “0”as absent. Data were entered into a (0, 1) matrix. The cluster analysis between glutenin subunit compositions were performed using the R method.

According to Liu’s calculation method, the following genetic diversity indicators were calculated: alleles per locus (A), percent polymorphic loci (P) (≤0.99 criterion), effective number of alleles per locus (Ae), and expected heterozygosity (He), to measure the genetic diversity of the population (Liu *et al.* 2007).

The Nei (1973) calculation method was used to calculate total genetic diversity (*H*_t_), genetic diversity within populations (*H*_s_), genetic diversity among populations (*D*_st_), proportion of genetic variation occurring among populations (*G*_st_) and interpopulational gene diversity relative to the intrapopulational gene diversity (*R*_st_), and evaluate the degree of genetic differentiation among populations and in the population.

## Results

### Allelic variation at *Glu-1* locus

**Fig. 1.**
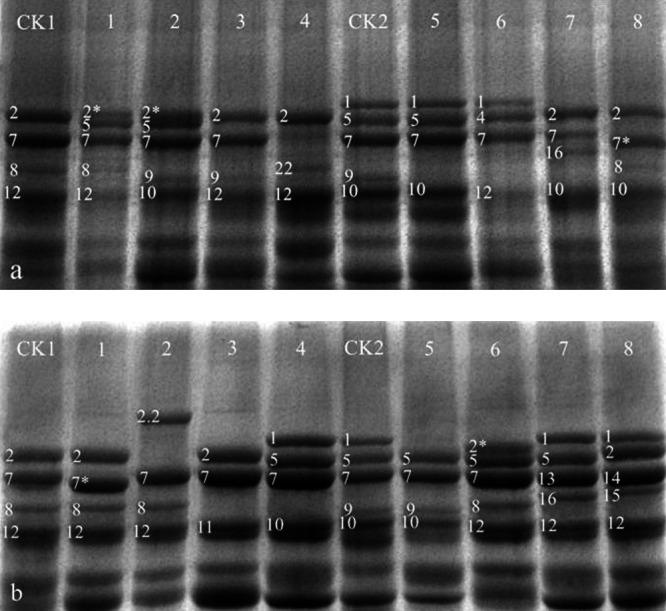
SDS-PAGE patterns some rare HMW-GS from Chinese wheat landraces (a) and commercial varieties (b). Wheat landraces (a), lanes are as follow: (CK1) Chinese Spring; (1) Pin II-4; (2) Qianjiaomai; (3) Baiyoumai; (4) Mujiadanga; (CK2) Zhongyou 9507; (5) Maogan; (6) Yangmai; (7) Wujiangcao; (8) Banjiemai. Commercial varieties (b), lanes are as follow: (CK1) Chinese Spring; (1) Cang 6003; (2) Jinuo 200; (3) Xiaoshan 8 hao; (4) Han 10-5223; (CK2) Zhongyou 9507; (5) Chuanshuangmai 1 hao; (6) Pingyang 56; (7) Hanmai 18 hao; (8) Han 6172.

Amongst the 1523 wheat lines examined, a total of 26 different HMW-GS alleles at the *Glu-1* were detected: three at *Glu-A1*, fourteen at *Glu-B1* and nine at *Glu-D1* (Table 1), of which 23, 19, 18, 14, 12 and 17 different alleles at the *Glu-1* occurred in 597 HWWR and MLYWWR landraces, 635 HWWR and MLYWWR commercial varieties, 504 HWWR commercial varieties, 131 MLYWWR commercial varieties, 133 SWWR commercial varieties, and 158 NSWR commercial varieties, respectively. An example of HMW-GS patterns in some landraces and commercial varieties is shown in Fig. 1.

At the *Glu-A1* locus, three subunits were detected: subunits 1, 2*, and Null. Among them, the *Glu-A1a* (subunit 1) and *Glu-A1b* (subunit 2*) alleles were better- bread-making quality subunits comparing with *Glu-A1c* (subunit Null). The frequency distribution for the subunits 1, 2*, and Null in the 597 landraces were 16.08%, 6.20%, and 77.72%, respectively, and were 41.73%, 2.36%, and 55.91% in the 635 HWWR and MLYWWR commercial varieties, respectively. With the same order, the three subunits had the respective frequencies of 44.09%, 22.28%, and 34.63% in all commercial varieties. Among the HWWR varieties, the three subunits had frequencies of 42.66%, 2.18%, and 55.16%. Similarly, for the MLYWWR varieties, the Null subunit had the highest frequencies of 58.78% while 1 and 2* had 38.17 % and 3.05%, respectively. In SWWR varieties, subunits 1, 2*, and Null had frequencies of 43.61%, 0.75% and 55.64%, respectively. Interestingly, for the NSWR varieties, the subunit 1 had a frequency of 48.73%, being the most abundant in the commercial cultivars, while subunits 2* and Null were 6.33% and 44.94%, respectively (Table 1).

At the *Glu-B1* locus, fourteen HMW-GS alleles were detected from the landraces and commercial cultivars. The landraces contained thirteen alleles, while the commercial varieties from the same regions (HWWR and MLYWWR) only had nine alleles. The subunit combinations or alleles 7, 7+8, 7+9, 14+15 and 17+18 were appeared in both landraces and commercial cultivars. For the landraces, alleles 7+8 and 7+9 were present at frequencies of 74.04% and 11.73%, respectively, and rare alleles 22, 13+19, 7+16 and 7+22 were detected with a frequency of 0.17% for both. Among the HWWR and MLYWWR commercial cultivars, alleles 7+8 and 7+9 had frequencies of 41.42% and 44.59%, respectively. Allele 14+15 was found in the landraces (1.51%) and the same region commercial cultivars (HWWR and MLYWWR, 8.03%) (Table 1). There were 8, 6, 7, and 9 different *Glu-B1* alleles detected in cultivars of HWWR, MLYWWR, SWWR and NSWR, respectively. Alleles 7+8 and 7+9 had the highest frequencies in four cultivation regions, including HWWR (36.71%, 48.61%), MLYWWR (59.54%, 29.01%), SWWR (28.57%, 30.08%) and NSWR (60.76%, 24.05%). Rare subunits were found in the three wheat growing regions, including MLYWWR (subunit 20, 2.29%), HWWR (7*+8, 0.40%) and NSWR (20X+20Y, 0.63%). Subunit 13+16 was detected in regions HWWR, SWWR and NSWR with frequencies of 2.18%, 4.51% and 1.27%, respectively. The alleles 14+15 and 17+18 were also frequently appeared, with frequencies of 15.04% and 6.77% in SWWR, respectively. The allele 6+8 was appeared with a high frequency of 15.53% in SWWR and also in HWWR and NSWR cultivars with lower frequencies (Table 1).

Seven alleles were found at the *Glu-D1* locus. Five alleles including 2+10, 4+12, 5+12, 5+10 and 5+12 were observed among the landraces and the same region commercial varieties. Allele 2+12 was detected most frequently in landraces (88.61%) and the commercial varieties (60.47%). The high quality alleles 5+10 and 5+12 had the respective frequencies of 7.71% and 0.34% in the landraces and 35.28% and 1.57% in the same region commercial varieties. Allele 2+10 and 4+12 were observed in the landraces (1.84% and 0.85%, respectively) and commercial varieties (1.73% and 0.47%, respectively). Alleles 3+12 and Null appeared with low frequencies in the landraces (0.17% and 0.5%, respectively), while alleles 2.2+12 and 2+11 were observed among the same region commercial varieties (0.31% and 0.16%, respectively) (Table 1). The higher frequencies of subunits 2+12 and 5+10 were detected in commercial cultivars from four regions, including HWWR (59.92%, 35.71%), MLYWWR (62.60%, 33.59%), SWWR (55.64%, 44.36%) and NSWR (62.03%, 30.38%). Alleles 4+12 and 2.2+12 were observed in HWWR cultivars (0.4%, 0.76%) and MLYWWR cultivars (0.2%, 0.79%). The frequencies of allele 2+10 were 1.59%, 2.29% and 1.27% in HWWR, MLYWWR and NSWR cultivars, respectively. Rare alleles 5+12 and Null were observed in HWWR cultivars (5+12, 1.98%) and NSWR cultivars (Null, 0.63%), respectively, and subunit 2+11 was detected in HWWR cultivars (0.20%) and NSWR cultivars (5.70%) (Table 1).

### Frequencies of HMW-GS compositions at the *Glu-1* loci

A total of 65 allelic combinations were observed at the three *Glu-1* loci from 597 landraces and 635 same region commercial varieties (Table 2). The *Glu-1* quality scores (Payne *et al.* 1987) of the wheat landraces and commercial varieties ranged from 4 to 10. However, the quality scores of 34 landraces and 17 commercial varieties were not determined due to the presence of rare subunits. Allelic combination “null, 7+8, and 2+12” appeared most frequently in landraces (56.28%), followed by three combinations including “1, 7+8, 2+12”, “2*, 7+8, 2+12”, “null, 7+9, 2+12” with frequencies of 7.87%, 3.85% and 5.70%, respectively. A total of 45 allelic combinations at the *Glu-1* loci were observed in the HWWR and MLYWWR commercial varieties. Of these, two combinations including null, 7+8, and 2+12, and null, 7+9, and 2+12 were clearly dominant in the commercial varieties with frequencies of 16.54% and 18.74%, respectively. Six other allelic combinations from the HWWR and MLYWWR commercial varieties appeared in low frequencies, including “1, 7+8, 2+12” (6.46%), “1, 7+8, 5+10” (7.87%), “1, 7+9, 2+12” (10.55%), “1, 7+9, 5+10” (7.56%), “null, 7+8, 5+10” (6.93%) and “null, 7+9, 5+10” (5.35%). The remaining combinations were observed from the landraces and the same region commercial varieties with frequencies between 0.16% and 2.99% (Table 2).

A total of 58 allelic combinations were observed in 4 commercial wheat cultivation regions (Table 3), including 40, 23, 26 and 33 combinations in the HWWR, MLYWWR, SWWR and NSWR commercial varieties, respectively. Three combinations “1, 7+9, 2+12”, “null, 7+8, 2+12” and “null, 7+9, 2+12” appeared as the most frequent among HWWR, with frequencies of 12.30% 14.29% and 21.38%, respectively, followed by three combinations “1, 7+8, 5+10”, “1, 7+9, 5+10” and “null, 7+8, 5+10” with frequencies of 8.53%,7.94% and 6.55%, respectively. The frequencies of the remaining combinations were less than 5%. Allelic combinations “1, 7+8, 2+12” and “null, 7+8, 2+12” were the most frequent in MLYWWR (14.50%, 25.19%) and NSWR (21.52%, 18.35%), while the frequencies of “null, 7+8, 5+10”, “null, 7+9, 2+12” and “null, 7+9, 5+10” were 8.40%, 9.16% and 8.40% in MLYWWR, respectively. The combinations “1, 7+8, 5+10”, “1, 7+9, 2+12”, and “null, 7+9, 2+12” occurred at frequencies of 9.49%, 6.33%, and 9.49% in MLYWWR, respectively. The remaining combinations were of low frequency in MLYWWR and NSWR. One combination “null, 7+9, 2+12” was most frequent (14.29%) in SWWR cultivars while the combinations “1, 14+15, 2+12”, “null, 7+8, 2+12” and “null, 7+8, 5+10” occurred with relatively high frequencies of 7.52%, 9.02%, 9.77%, respectively (Table 3).

### Cluster analysis of HMW-GS compositions in different wheat planting regions

**Fig. 2.**
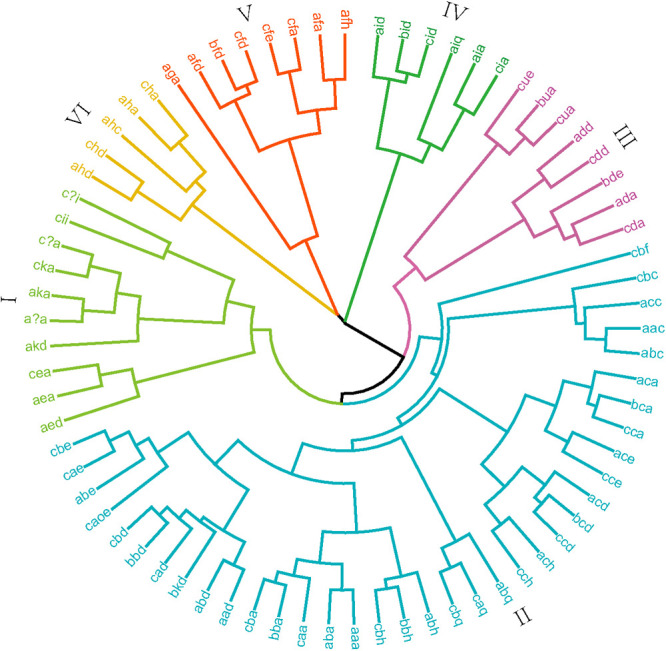
Cluster analysis based on allele combinations at the *Glu-1* loci of Chinese wheat landraces and commercial varieties.

In order to determine the difference in the proportion of high-quality allele combinations between different wheat planting regions, 73 allele combinations were used to perform a cluster analysis on all varieties. Rare combination “null, 20X+20Y, null” was not used for cluster analysis. Cluster analysis based on allelic similarity at the *Glu-1* loci classified varieties into six major categories (Fig. 2). Calculating the quality scores of the allelic combinations, the category II ranged from 4 to 10. The quality scores of categories IV, V and VI ranged from 6 to 10 with most allelic combinations above 8. Due to rare allelic combinations, the quality score was not calculated in categories I and III. The category II consists of 88.15% of the landraces and 87.22% of the same region commercial varieties. Only 6.92% of the landraces and 2.04% of the same region commercial varieties fall in categories I+III. Categories IV+V+VI contain 5.38% of the landraces and 10.55% of the same region commercial varieties, of which most of the cultivars had high quality scores. The landraces had lower genetic diversity and proportion of high-quality subunit combinations than these of the same region commercial varieties.

Cluster analysis of allelic combinations generated six categories, suggesting that significant variations exist in the HMW compositions among the four wheat regions (Fig. 2). Most commercial cultivars from the four regions fell into the category II. While the IV+V+VI categories contained 11.53%, 6.86%, 26.3% and 5.68% of the HWWR, MLYWWR, SWWR and NSWR cultivars, respectively. Only 1.99%, 2.29%, 13.53% and 6.32% of the commercial varieties in HWWR, MLYWWR, SWWR and NSWR belong to categories I+III, respectively. These results indicate that the genetic diversity of the SWWR cultivars was the highest, while the lowest was MLYWWR. The SWWR cultivars had the highest proportion of high-quality subunit combinations while the NSWR had the lowest.

### Genetic variation at the *Glu-1* loci within and between populations

According to the germplasm originating sites, the research materials are divided into six populations, including four commercial cultivar populations from HWWR, MLYWWR, SWWR and NSWR, one landrace population and one commercial cultivar population come from HWWR and MLYWWR. The genetic differentiation was analyzed within and between populations. The values of genetic differentiation parameters of the HMW-GS locus of the six populations were presented in Table 4. When the four individual commercial variety populations combined together, the genetic diversity indicators were calculated as A (alleles per locus) = 7.33, P (percentage of polymorphic loci) = 1.00, Ae (effective number of alleles per locus) = 2.347 and He (mean diversity index) = 0.563, and the average values of the four regions were A = 5.09, P = 1.00, Ae = 2.349 and He = 0.552 (Table 4). The largest A value was 6.00 in the HWWR population. The P value for every population was 1.00. The Ae and He were 2.841 and 0.591 in the SWWR population, respectively. Among the four commercial region populations, the highest genetic diversity was found in the SWWR population (A = 4.00, P = 1.00, Ae = 2.841 and He = 0.591), while the MLYWWR population showed the lowest (A = 4.67, P = 1.00, Ae = 2.090 and He = 0.520). The genetic diversity of the landraces plus the same region commercial varieties was A = 8.33, P = 1.00, Ae = 1.994 and He = 0.486, and the average values of the landrace population and the same region commercial variety population was A = 7.00, P = 1.00, Ae = 1.892 and He = 0.442 (Table 4). In comparison with the landrace population and the same region commercial variety population, the former had A = 6.33, P = 1.00, Ae = 2.247 and He = 0.548, which was greater than that of the latter population (A = 7.67, P = 1.00, Ae = 1.537 and He = 0.337). This result was similar to the result of cluster analysis, mainly as the frequency of allele distribution within the commercial variety population was relatively uniform, and the distribution was relatively concentrated in the landrace population.

Genetic differentiation at the *Glu-1* loci in different wheat regions are presented in Table 5. When all commercial wheat varieties were analyzed together, the average total genetic diversity (*H*_t_) for the three loci was 0.563 (*Glu-A1* at 0.521, *Glu-B1* at 0.656 and *Glu-D1* at 0.511). The mean genetic diversity of each population (*H*_s_ = 0.552) was much higher than the mean genetic diversity among populations (*D*_st_ = 0.011), indicating that the genetic diversity between populations was lower than that within populations. The average relative differentiation among populations was *G*_st_ = 0.017 and ranged from 0.002 at *Glu-A1* to 0.024 at *Glu-B1*, indicating that 1.7% of the gene diversity was among populations while 98.3% of the gene diversity was within populations.

The average total genetic diversity (*H*_t_) of the *Glu-1* loci was 0.486 in the landraces combining the same region commercial varieties, the mean genetic diversity within populations (*H*_s_) and mean genetic diversity among populations (*D*_st_) was 0.442 and 0.044, respectively. This shows that the genetic diversity between the landrace population and the same region commercial variety population was lower than that within these two wheat populations. The average relative differentiation between populations was *G*_st_ = 0.091, indicating 9.1% of the gene diversity was between the two populations while 90.9% of the gene diversity was within populations.

## Discussion

Utilization of gluten allelic variation in breeding programs is the key to improving wheat quality (Ma *et al.* 2019). Twenty-six different allelic variations at the *Glu-1* loci were found among 1523 wheat lines in the current study. Among them, 23 and 22 different HMW-GS alleles were detected in landraces and commercial varieties, respectively. According to previous reports, Zheng *et al.* (2011) found 22 HMW-GS alleles in studies involving 485 landraces from the Yangtze River region. Liu *et al.* (2007) identified 16 HMW-GS alleles in 111 Hubei wheat landraces. Zhang *et al.* (2002) detected 28 HMW glutenin alleles from 3,459 Chinese landraces. Liu *et al.* (2005) reported 16 alleles in 251 Chinese commercial cultivars. More recently, Dai *et al.* (2020) detected 16 alleles at the *Glu-1* loci in 300 Xinjiang wheat landraces. Gao *et al.* (2020) found 15 allelic variations at the *Glu-1* loci in commercial cultivars from China. In comparison, Yasmeen *et al.* (2015) detected much higher number of alleles at *Glu-1* in indigenous landraces and commercial cultivars of Pakistan.

Previous reports showed that subunits 1 or 2* at the *Glu-A1* locus have better effects to improve bread-making quality than null subunit (Luo *et al.* 2001). The frequency of other *Glu-A1* alleles in durum wheat landraces have shown higher than that of null allele (Ammar *et al.* 2000, Branlard *et al.* 1989, Magallanes-López *et al.* 2017). In some reports, the null allele was the most frequent at the *Glu-A1* locus for Chinese wheat landrace (Dai *et al.* 2020, Wei *et al.* 2000, Yan *et al.* 2007, Zheng *et al.* 2011). In this study, the null allele was observed to be the most frequent at *Glu-A1* in wheat landraces (77.72%) and the same region commercial cultivars (55.91%). Whereas 41.73% of the commercial varieties contains the subunit 1, far higher than that of the landraces (16.08%). The null allele was observed in a high frequency in three regions (HWWR, MLYWWR and SWWR). In NSWR, the frequency order is 1 > null > 2*, which is consistent with the results of Liu *et al.* (2005). The high frequency of the *Glu-A1a* had been observed in Spanish (86.5%) and European (84.81%) wheat varieties (An *et al.* 2005, Caballero *et al.* 2004). Yasmeen *et al.* (2015) showed that the 2* and null alleles were the most frequent in Pakistan wheat varieties and landraces. For the *Glu-B1* locus, 14 and 11 alleles were detected in the landraces and commercial cultivars, respectively. The most frequent alleles were 7+8 and 7+9. The frequency of 7+8 was 74.04% in the landraces, which is consistent with the results of other Chinese and Japanese landraces (Dai *et al.* 2020, Liu *et al.* 2007, Nakamura 2000, Zheng *et al.* 2011). In contrast, the major allele in India and Pakistan landrace was 17+18 (Goel *et al.* 2018, Niwa *et al.* 2008). Two alleles, *Glu-B1b* (7+8) and *Glu-B1c* (7+9) appeared most frequently in four commercial wheat cultivation regions. Liu *et al.* (2005) identified 7+8 (*Glu-B1b*) and 7+9 (*Glu-B1c*) were the major alleles in Chinese commercial cultivars. Alleles (*Glu-B1b*) 7+8 and (*Glu-B1c*) 7+9 were predominant in varieties from France, Argentina and Pakistan (Branlard *et al.* 1989, Lerner *et al.* 2009, Tabasum *et al.* 2011), whereas alleles 13+16 (*Glu-B1f*) was the most frequent in varieties from Spanish (An *et al.* 2005, Caballero *et al.* 2004). Liu *et al.* (2007) found that the *Glu-B1h* (14+15) allele was a unique allele in Chinese wheat cultivars, which was reported to enhance the dough quality parameters such as SDS sedimentation value and resistance breakdown value (Brites and Carrillo 2001). In the current study, the *Glu-B1d* (6+8) allele frequency was higher in SWWR than that of other Chinese wheat Regions. Subunits 7 and 17+18 were both identified with low frequency in the landraces and commercial cultivars. Allele 17+18 (*Glu-B1i*) had positive effect on sedimentation and mixograph (Carrillo *et al.* 1990, Ram 2003). Alleles *Glu-B1ao* (7+16) and *Glu-B1g* (13+19) occurred at lower frequencies in the landraces and were not detected in four commercial wheat growing regions.

Allelic variations at the *Glu-D1* locus were important for dough quality (Kolster *et al.* 1991). Gupta *et al.* (1994) showed that allele *Glu-D1d* (5+10) is associated with superior bread-making quality while allele *Glu-D1a* (2+12) reduces the bread-making quality. In the current study, the frequency of the *Glu-D1a* (2+12) allele was far higher in the landraces than commercial cultivars. Previous reports also showed that the allele *Glu-D1a* (2+12) was the most frequent in Chinese and Japanese wheat landraces (Dai *et al.* 2020, Liu *et al.* 2007, Nakamura 2000, Zhang *et al.* 2002, Zheng *et al.* 2011). For commercial cultivars, allele *Glu-D1a* (2+12) also appeared more than allele *Glu-D1d* (5+10) although the latter allele also appeared as a common allele, which is in agreement with previous results (Gao *et al.* 2018, 2020, Liu *et al.* 2005). According to Yasmeen *et al.* (2015), allele *Glu-D1d* (5+10) was the most frequent in Pakistan commercial wheat varieties. It is worth noting that in our study we detected rare subunits 2.2+12 and 2+11 among commercial wheat cultivars. Nakamura (1999) showed that the subunit 2.2+12 was found frequently in Japanese varieties. Meanwhile, allele *Glu-D1h* (5+12) was detected in our study, which was reported to have shown better overall quality characteristics and bread loaf volume in synthetic hexaploids (Peña *et al.* 1995). Overall, the frequency of subunits *Glu-D1d* (5+10) and *Glu-D1h* (5+12) were higher in the Chinese commercial wheat cultivars than wheat landraces in our study.

For HMW-GS compositions, “null, 7+8, 2+12” was the most frequent (56.28%) allelic combination for wheat landraces which is consistent with previous studies (Dai *et al.* 2020, Liu *et al.* 2007, Wei *et al.* 2000, Zheng *et al.* 2011). Nakamura (2000) reported that it was the most frequent among wheat landraces from Japan. However, different frequencies of allelic combinations were observed among different regions. The genotypes “null, 7+9, 2+12” appeared as the most frequent in HWWR (21.23%) and SWWR (14.29%), while “null, 7+8, 2+12” and “1, 7+8, 2+12” occurred at frequencies of 25.19% and 21.52% in MLYWWR and NSWR, respectively. Gao *et al.* (2018) reported that “null, 7+9, 2+12” was most frequent in 151 Hebei winter wheat varieties. Our results were similar to that reported by Zhang *et al.* (2002).

The allele combinations in the current study were classified into six categories, and the proportion of high-quality allele combinations of wheat commercial varieties was greater than that of the landraces. The average *Glu-1* quality score of Chinese landraces were similar to that of the landraces from Japan, but lower than that of the landraces of Pakistan (Nakamura 2000, Yasmeen *et al.* 2015). Other results showed that the genetic diversity and proportion of high-quality subunit combinations of the SWWR region was most abundant among the four regions. The high value of genetic diversity means the high proportion of rare alleles (Novoselskaya-Dragovich *et al.* 2011). High-quality alleles *Glu-B1i* (17+18), *Glu-B1f* (13+16) and *Glu-B1h* (14+15) were found in the IV, V and VI categories, which occurred at a high frequency of 26.3% in SWWR. However, the highest proportion of allele combinations of 10 quality scores were detected in HWWR and NSWR. HWWR and NSWR are major noodle-consuming area, resulting in a relatively high frequency of the high-quality subunit combinations. The frequencies of the 10 quality score allele combinations were far higher in the commercial cultivars than in the landraces, indicating the impact of modern breeding programs in China. The average *Glu-1* loci quality scores of commercial wheat varieties in China was below average in global aspects. For example, Novoselskaya-Dragovich *et al.* (2011) reported that the average *Glu-1* loci quality score of Chinese wheat commercial varieties was similar to that of the wheat varieties from Italy but were less than those from Russia and Canada.

The level of genetic diversity indicators for HMW-GS found in the four commercial wheat regions was He = 0.563, with the SWWR population showing the highest (He = 0.591) and the MLYWWR population showing the lowest (He = 0.520). Our results were similar to that reported by Zhang *et al.* (2002). Novoselskaya-Dragovich *et al.* (2011) found a similar genetic diversity index in Chinese wheat cultivars, which was higher than these of the Canada and England lines but lower than these of the French, Italy and Australia bread wheats. The genetic diversity index in all Chinese commercial cultivars was higher than that of the Argentinean wheats (He = 0.458) (Lerner *et al.* 2009). The genetic diversity index of the wheat landraces was 0.337, lower than that of the same region commercial cultivars (0.548) but higher than that of the Chinese core collection (0.232) (Zhang *et al.* 2002) and Hubei landraces (0.238) (Liu *et al.* 2007). Nakamura (2000) found a lower genetic diversity in the Japanese landraces (He = 0.265) (Ruiz *et al.* 2002). The number of alleles per locus (A) of the landrace population was 7.67, which was higher than that of the same region commercial cultivars (6.33). The results indicated that the distribution of variation types of Chinese commercial varieties was more balanced than that of the landraces. Due to self-pollination and natural isolation of wheat, gene exchange between populations was very limited, resulting in some alleles only exist in a relatively narrow range. In the commercial varieties, the introduction of artificial hybridization promoted the exchange of genetic information between different countries and ecological regions, which significantly improved the genetic diversity of the breeding varieties (Novoselskaya-Dragovich *et al.* 2011). As a matter of fact, 1.7% of the gene diversity was among the four regions while 98.3% was within regions. This result proves that the allele types selected by breeding in the four major regions are similar.

The increase of genetic diversity in commercial varieties from the four Chinese wheat regions may be due to the increase of the proportion of high-quality subunits in breeding programs. Due to high allele diversity, the Chinese landraces have the potential to be used wheat quality breeding in China.

A large number of new subunit combinations exist in landraces, which did not exist in commercial varieties. Cluster analysis of the allelic combinations showed that the proportion of high-quality subunit combinations was higher in commercial varieties than that of the landraces. The rare alleles found in landraces indicated that the Chinese landraces can potentially useful for wheat quality improvement. The genetic diversity analysis revealed limited subunit combinations in Chinese commercial wheat varieties, especially in the southwest winter wheat region.

## Author Contribution Statement

X. W., R. S., Y. A., H. P. and S. G. conducted the experiments measurements. X. R. conceived this study and designed the experiments. X. W. performed the statistical analysis and wrote the manuscript. X. R. and D. S. coordinated the experiments and oversaw the data analysis. X. R. revised the manuscript. All authors had read and approved the final version of the manuscript.

## Supplementary Material

Supplemental Table

## Figures and Tables

**Table 1.** Allelic variation and frequency at the *Glu-1* loci of Chinese wheat landraces and commercial varieties

Loci	Allele	Subunit	Landraces (HWWR and MLYWWR)		Commercial varieties (HWWR and MLYWWR)		Commercial varieties (HWWR)		Commercial varieties (MLYWWR)		Commercial varieties (SWWR)		Commercial varieties (NSWR)	
			NO	(%)	NO	(%)	NO	(%)	NO	(%)	NO	(%)	NO	(%)
*Glu-A1*	a	1	96	16.08	265	41.73	215	42.66	50	38.17	58	43.61	77	48.73
	b	2*	37	6.20	15	2.36	11	2.18	4	3.05	1	0.75	10	6.33
	c	N	464	77.72	355	55.91	278	55.16	77	58.78	74	55.64	71	44.94
*Glu-B1*	a	7	11	1.84	9	1.42	6	1.19	3	2.29	2	1.50	3	1.90
	e	20	8	1.34	3	0.47	–	–	3	2.29	–	–	–	–
	k	22	1	0.17	–	–	–	–	–	–	–	–	6	3.80
	f	13+16	19	3.18	11	1.73	11	2.18	–	–	6	4.51	2	1.27
	g	13+19	1	0.17	–	–	–	–	–	–	–	–	–	–
	h	14+15	9	1.51	51	8.03	44	8.73	7	5.34	20	15.04	1	0.63
	i	17+18	4	0.67	5	0.79	3	0.60	2	1.53	9	6.77	6	3.80
	d	6+8	7	1.17	8	1.26	8	1.59	–	–	18	15.53	5	3.16
	u	7*+8	17	2.85	2	0.31	2	0.40	–	–	–	–	–	–
	ao	7+16	1	0.17	–	–	–	–	–	–	–	–	–	–
	?	7+22	7	1.17	–	–	–	–	–	–	–	–	–	–
	b	7+8	442	74.04	263	41.42	185	36.71	78	59.54	38	28.57	96	60.76
	c	7+9	70	11.73	283	44.57	245	48.61	38	29.01	40	30.08	38	24.05
	?	20X+20Y	–	–	–	–	–	–	–	–	–	–	1	0.63
*Glu-D1*	e	2+10	11	1.84	11	1.73	8	1.59	3	2.29	–	–	2	1.27
	a	2+12	529	88.61	384	60.47	302	59.92	82	62.60	74	55.64	98	62.03
	b	3+12	1	0.17	–	–	–	–	–	–	–	–	–	–
	c	4+12	5	0.84	3	0.47	2	0.40	1	0.76	–	–	–	–
	d	5+10	46	7.71	224	35.28	180	35.71	44	33.59	59	44.36	48	30.38
	h	5+12	2	0.34	10	1.57	10	1.98	–	–	–	–	–	–
	i	N	3	0.50	–	–	–	–	–	–	–	–	1	0.63
	f	2.2+12	–	–	2	0.31	1	0.20	1	0.79	–	–	–	–
	q	2+11	–	–	1	0.16	1	0.20	–	–	–	–	9	5.70

HWWR: Huang-huai Winter Wheat Region, MLYWWR: Middle and Low Yangtze Winter Wheat Region, SWWR: Southwest Winter Wheat Region, NSWR: Northwest Spring Wheat Region.

**Table 2.** High-molecular-weight glutenin subunit compositions and *Glu-1* quality score of Chinese wheat landraces and commercial varieties

HMW-GS compositions			Alleles*^a^*	Total (1232)	Landraces (HWWR and MLYWWR) (597)		Commercial varieties (HWWR and MLYWWR) (635)		*Glu-1* quality score
*Glu-A1*	*Glu-B1*	*Glu-D1*		NO	NO	(%)	NO	(%)	
1	13+16	2+12	afa	2	2	0.34	–	–	8
1	13+16	5+10	afd	6	1	0.17	5	0.79	10
1	13+16	5+12	afh	1	–	–	1	0.16	10
1	14+15	2+12	aha	19	–	–	19	2.99	8
1	14+15	4+12	ahc	1	–	–	1	0.16	7
1	14+15	5+10	ahd	13	–	–	13	2.05	10
1	13+19	2+12	aga	1	1	0.17	–	–	?
1	17+18	2+12	aia	1	–	–	1	0.16	8
1	17+18	5+10	aid	3	–	–	3	0.47	10
1	20	2+12	aea	2	2	0.34	–	–	6
1	20	5+10	aed	3	–	–	3	0.47	8
1	6+8	2+12	ada	1	1	0.17	–	–	6
1	6+8	5+10	add	3	2	0.34	1	0.16	8
1	7	2+12	aaa	5	1	0.17	4	0.63	6
1	7	4+12	aac	1	1	0.17	–	–	5
1	7	5+10	aad	3	2	0.34	1	0.16	8
1	7+22	2+12	a?a	1	1	0.17	–	–	?
1	7+8	2+10	abe	1	1	0.17	–	–	?
1	7+8	2+12	aba	88	47	7.87	41	6.46	8
1	7+8	4+12	abc	2	1	0.17	1	0.16	7
1	7+8	5+10	abd	61	11	1.84	50	7.87	10
1	7+8	5+12	abh	1	–	–	1	0.16	?
1	7+9	2+10	ace	2	1	0.17	1	0.16	?
1	7+9	2+12	aca	83	16	2.68	67	10.55	7
1	7+9	4+12	acc	1	1	0.17	–	–	6
1	7+9	5+10	acd	52	4	0.67	48	7.56	9
1	7+9	5+12	ach	3	1	0.17	2	0.31	9
2*	13+16	5+10	bfd	2	1	0.17	1	0.16	10
2*	7*+8	2+12	bua	2	2	0.34	–	–	?
2*	7+8	2+12	bba	23	23	3.85	–	–	8
2*	7+8	5+10	bbd	9	5	0.84	4	0.63	10
2*	7+8	5+12	bbh	1	–	–	1	0.16	10
2*	7+9	2+12	bca	7	3	0.50	4	0.63	7
2*	7+9	5+10	bcd	8	3	0.50	5	0.79	9
N	13+16	2+10	cfe	1	1	0.17	–	–	?
N	13+16	2+12	cfa	14	12	2.01	2	0.31	6
N	13+16	5+10	cfd	4	2	0.34	2	0.31	8
N	14+15	2+12	cha	19	9	1.51	10	1.57	6
N	14+15	5+10	chd	8	–	–	8	1.26	8
N	17+18	2+12	cia	4	3	0.50	1	0.16	6
N	17+18	N	cii	1	1	0.17	–	–	?
N	20	2+12	cea	6	6	1.01	–	–	4
N	22	2+12	cka	1	1	0.17	–	–	?
N	6+8	2+12	cda	7	2	0.34	5	0.79	4
N	6+8	5+10	cdd	4	2	0.34	2	0.31	6
N	7*+8	2+10	cue	1	1	0.17	–	–	?
N	7*+8	2+12	cua	16	14	2.35	2	0.31	?
N	7	2+10	cae	1	1	0.17	–	–	?
N	7	2+11	caq	1	–	–	1	0.16	?
N	7	2+12	caa	8	6	1.01	2	0.31	4
N	7	5+10	cad	1	–	–	1	0.16	6
N	7+16	2+10	caoe	1	1	0.17	–	–	?
N	7+22	2+12	c?a	4	4	0.67	–	–	?
N	7+22	N	c?i	2	2	0.34	–	–	?
N	7+8	2.2+12	cbf	2	–	–	2	0.31	?
N	7+8	2+10	cbe	14	5	0.84	9	1.42	?
N	7+8	2+12	cba	441	336	56.28	105	16.54	6
N	7+8	4+12	cbc	4	3	0.50	1	0.16	5
N	7+8	5+10	cbd	54	10	1.68	44	6.93	8
N	7+8	5+12	cbh	5	1	0.17	4	0.63	8
N	7+9	2+10	cce	3	2	0.34	1	0.16	?
N	7+9	2+12	cca	153	34	5.70	119	18.74	5
N	7+9	5+10	ccd	37	3	0.50	34	5.35	7
N	7+9	5+12	cch	3	1	0.17	2	0.31	7

HWWR: Huang-huai Winter Wheat Region, MLYWWR: Middle and Low Yangtze Winter Wheat Region. *^a^* Alleles were subunit combinations of *Glu-A1*, *Glu-B1* and *Glu-D1* loci, for example, “afa” allele composition was defined “the first a = 1 subunit at *Glu-A1*, f = 13+16 subunit at *Glu-B1*, the second a = 2+12 subunit at *Glu-D1*”, and the “afd” allele composition was defined “a = 1 subunit at *Glu-A1*, f = 13+16 subunit at *Glu-B1*, d = 5+10 subunit at *Glu-D1*”.

**Table 3.** High-molecular-weight glutenin subunit compositions and *Glu-1* quality score of Chinese four commercial wheat cultivation regions

HMW-GS compositions			Alleles*^a^*	Total (926)	HWWR (504)		MLYWWR (131)		SWWR (133)		NWSWR (158)		*Glu-1* quality score
*Glu-A1*	*Glu-B1*	*Glu-D1*		NO	NO	(%)	NO	(%)	NO	(%)	NO	(%)	
1	13+16	2+12	afa	3	–	–	–	–	2	1.50	1	0.63	8
1	13+16	5+10	afd	6	5	0.99	–	–	1	0.75	–	–	10
1	13+16	5+12	afh	1	1	0.20	–	–	–	–	–	–	10
1	14+15	2+12	aha	30	15	2.98	4	3.05	10	7.52	1	0.63	8
1	14+15	4+12	ahc	1	1	0.20	–	–	–	–	–	–	7
1	14+15	5+10	ahd	18	13	2.58	–	–	5	3.76	–	–	10
1	17+18	2+11	aiq	1	–	–	–	–	–	–	1	0.63	?
1	17+18	2+12	aia	2	–	–	1	0.76	–	–	1	0.63	8
1	17+18	5+10	aid	7	2	0.40	1	0.76	2	1.50	2	1.27	10
1	6+8	2+12	ada	7	–	–	–	–	7	5.26	–	–	6
1	6+8	5+10	add	8	1	0.20	–	–	6	4.51	1	0.63	8
1	7	2+12	aaa	5	3	0.60	1	0.76	1	0.75	–	–	6
1	7	5+10	aad	1	1	0.20	–	–	–	–	–	–	8
1	7+8	2+11	abq	2	–	–	–	–	–	–	2	1.27	?
1	7+8	2+12	aba	81	22	4.37	19	14.50	6	4.51	34	21.52	8
1	7+8	4+12	abc	1	1	0.20	–	–	–	–	–	–	7
1	7+8	5+10	abd	72	43	8.53	7	5.34	7	5.26	15	9.49	10
1	7+8	5+12	abh	1	1	0.20	–	–	–	–	–	–	10
1	7+9	2+10	ace	1	1	0.20	–	–	–	–	–	–	?
1	7+9	2+12	aca	83	62	12.30	5	3.82	6	4.51	10	6.33	7
1	7+9	5+10	acd	58	40	7.94	8	6.11	5	3.76	5	3.16	9
1	7+9	5+12	ach	2	2	0.40	–	–	–	–	–	–	9
1	20	5+10	aed	3	–	–	3	2.29	–	–	–	–	8
1	22	2+12	aka	1	–	–	–	–	–	–	1	0.63	?
1	22	5+10	akd	3	–	–	–	–	–	–	3	1.90	?
2*	22	5+10	bkd	1	–	–	–	–	–	–	1	0.63	?
2*	13+16	5+10	bfd	1	1	0.20	–	–	–	–	–	–	10
2*	17+18	5+10	bid	1	–	–	–	–	–	–	1	0.63	10
2*	6+8	2+10	bde	1	–	–	–	–	–	–	1	0.63	?
2*	7+8	5+10	bbd	10	1	0.20	3	2.29	–	–	6	3.80	10
2*	7+8	5+12	bbh	1	1	0.20	–	–	–	–	–	–	10
2*	7+9	2+12	bca	4	3	0.60	1	0.76	–	–	–	–	7
2*	7+9	5+10	bcd	7	5	0.99	–	–	1	0.75	1	0.63	9
N	13+16	2+12	cfa	5	2	0.40	–	–	2	1.50	1	0.63	6
N	13+16	5+10	cfd	3	2	0.40	–	–	1	0.75	–	–	8
N	14+15	2+12	cha	14	7	1.39	3	2.29	4	3.01	–	–	6
N	14+15	5+10	chd	9	8	1.59	–	–	1	0.75	–	–	8
N	17+18	2+12	cia	4	1	0.20	–	–	2	1.50	1	0.63	6
N	17+18	5+10	cid	5	–	–	–	–	5	3.76	–	–	8
N	6+8	2+12	cda	10	5	0.99	–	–	3	2.26	2	1.27	4
N	6+8	5+10	cdd	5	2	0.40	–	–	2	1.50	1	0.63	6
N	7*+8	2+12	cua	2	2	0.40	–	–	–	–	–	–	?
N	7	2+11	caq	2	1	0.20	–	–	–	–	1	0.63	?
N	7	2+12	caa	3	1	0.20	1	0.76	–	–	1	0.63	4
N	7	5+10	cad	3	–	–	1	0.76	1	0.75	1	0.63	6
N	7+8	2.2+12	cbf	2	1	0.20	1	0.76	–	–	–	–	?
N	7+8	2+10	cbe	9	7	1.39	2	1.53	–	–	–	–	?
N	7+8	2+11	cbq	5	–	–	–	–	–	–	5	3.16	?
N	7+8	2+12	cba	146	72	14.29	33	25.19	12	9.02	29	18.35	6
N	7+8	4+12	cbc	1	–	–	1	0.76	–	–	–	–	5
N	7+8	5+10	cbd	61	33	6.55	11	8.40	13	9.77	4	2.53	8
N	7+8	5+12	cbh	4	3	0.60	1	0.76	–	–	–	–	8
N	7+9	2+10	cce	2	–	–	1	0.76	–	–	1	0.63	?
N	7+9	2+12	cca	153	107	21.23	12	9.16	19	14.29	15	9.49	5
N	7+9	5+10	ccd	50	23	4.56	11	8.40	9	6.77	7	4.43	7
N	7+9	5+12	cch	2	2	0.40	–	–	–	–	–	–	7
N	20X+20Y	N	c?i	1	–	–	–	–	–	–	1	0.63	?
N	22	2+12	cka	1	–	–	–	–	–	–	1	0.63	?

HWWR: Huang-huai Winter Wheat Region, MLYWWR: Middle and Low Yangtze Winter Wheat Region, SWWR: Southwest Winter Wheat Region, NSWR: Northwest Spring Wheat Region. *^a^* Alleles were subunit combinations of *Glu-A1*, *Glu-B1* and *Glu-D1* loci, for example, “afa” allele composition was defined “the first a = 1 subunit at *Glu-A1*, f = 13+16 subunit at *Glu-B1*, the second a = 2+12 subunit at *Glu-D1*”, and the “afd” allele composition was defined “a = 1 subunit at *Glu-A1*, f = 13+16 subunit at *Glu-B1*, d = 5+10 subunit at *Glu-D1*”.

**Table 4.** HMW subunits diversity at three *Glu-1* loci in populations of Chinese wheat landraces and commercial varieties

Population	Sample size	Alleles	A	P	Ae	He
HWWR	504	18	6.00	1.00	2.247	0.549
MLYWWR	131	14	4.67	1.00	2.090	0.520
SWWR	133	12	4.00	1.00	2.841	0.591
NSWR	158	17	5.67	1.00	2.218	0.548
Mean	–	–	5.09	1.00	2.349	0.552
Total	926	22	7.33	1.00	2.347	0.563
Landraces (HWWR and MLYWWR)	597	23	7.67	1.00	1.537	0.337
Commercial varieties (HWWR and MLYWWR)	635	19	6.33	1.00	2.247	0.548
Mean	–	–	7.00	1.00	1.892	0.442
Total	1232	25	8.33	1.00	1.994	0.486

HWWR: Huang-huai Winter Wheat Region, MLYWWR: Middle and Low Yangtze Winter Wheat Region, SWWR: Southwest Winter Wheat Region, NSWR: Northwest Spring Wheat Region.

**Table 5.** Differentiation of HMW subunits diversity within and between populations of Chinese wheat landraces and commercial varieties

Population	Locus	Alleles	*H*_t_	*H*_s_	*D*_st_	*G*_st_	*R*_st_
Four commercial wheat cultivation regions populations	*Glu-A1*	3	0.521	0.519	0.002	0.003	0.004
	*Glu-B1*	11	0.656	0.632	0.024	0.037	0.051
	*Glu-D1*	8	0.511	0.505	0.006	0.012	0.016
	Mean	–	0.563	0.552	0.011	0.017	0.024
Landraces (HWWR and MLYWWR) and Commercial varieties (HWWR and MLYWWR) populations	*Glu-A1*	3	0.470	0.439	0.031	0.066	0.141
	*Glu-B1*	13	0.587	0.529	0.058	0.099	0.219
	*Glu-D1*	9	0.402	0.359	0.043	0.108	0.241
	Mean	–	0.486	0.442	0.044	0.091	0.200

Four commercial wheat cultivation regions populations: Huang-huai Winter Wheat Region population, Middle and Low Yangtze Winter Wheat Region population, Southwest Winter Wheat Region population, Northwest Spring Wheat Region population. HWWR: Huang-huai Winter Wheat Region, MLYWWR: Middle and Low Yangtze Winter Wheat Region.
