# Supplementary material for: Allelic variation and genetic diversity of HMW glutenin subunits in Chinese wheat (Triticum aestivum L.) landraces and commercial cultivars
Source: Breed Sci. 2022 Feb 2;72(2):169–80. doi: 10.1270/jsbbs.21076 (PMC9522535; doi:10.1270/jsbbs.21076)
Supplement: Supplementary file 1 — Supplemental Table [file 72_169_s1.pdf]

doi: 10.1270/jsbbs.21076

Supplemental Table 1.The list of collected 1523 wheat varieties.

597 landraces from HWWR and MLYWWR		
Sampie ID	Common name	allelic combinations
L1	11C194	N/7+8/2+12
L2	pinⅡ-4	2*/7+8/5+10
L3	pin 6-Ⅲ	2*/7+8/5+10
L4	SN0052	N/13+16/5+10
L5	04 zhong 36	1/7+9/4+12
L6	pin11	N/7+8/2+12
L7	zhe mai 1	N/7+8/2+12
L8	bai huo mai	N/7+8/2+12
L9	D5611	1/7+8/5+10
L10	ge jia dou	N/7+8/2+12
L11	WA3D128	1/7+8/5+10
L12	15F7-4	1/7+9/2+10
L13	J64-4	1/7+8/2+10
L14	C-4	N/7+9/2+10
L15	da li ban zi	N/7+8/2+10
L16	gu lou ding	1/7+8/2+12
L17	pin Ⅱ-1	N/7+8/2+12
L18	bai tiao yu	N/7+8/2+12
L19	san yue huang	N/7+8/2+12
L20	pin 10	N/7+8/5+12
L21	pin bao	N/7+8/2+12
L22	bai bian sui	N/7+8/2+12
L23	WA2D27	N/7+8/2+12
L24	07K1312	N/7+8/2+12
L25	WA5(125)	N/7+8/2+12
L26	06 xuan	1/7+9/2+12
L27	Pin 7	1/7+9/5+10
L28	Huo Qiu	N/7+8/2+10
L29	Xin Shu Guang1	N/7+9/2+10
L30	En Mai 4	N/7/2+12
L31	Pin 6	N/17+18/2+12
L32	D1775-2	N/13+16/5+10
L33	Pin 5	N/7+8/5+10
L34	Guan 1	1/7+9/2+12
L35	zi Yuan Mai	N/7+8/2+12
L36	Feng Chan 3	N/7+8/2+12
L37	15F7-2	N/7+9/5+12
L38	J88-2	N/6+8/2+12
L39	Tu Mang Mai	N/7+8/2+12
L40	C1	1/7+9/5+10
L41	Xing Yi 4	N/7+9/2+12
L42	Dong Nong101	N/7+9/2+12
L43	Nuo MaiD5659	N/7+8/2+12

L44	Shu Wan 8	N/7+8/2+12
L45	Bai Hua Mai	1/7+8/2+12
L46	Ke Lao 4	1/7+9/2+12
L47	wen mai 6	1/7+9/5+10
L48	gamai	N/7+8/2+12
L49	jing yang 60	N/7+8/2+12
L50	Chong Bai Zi Mai	1/7+8/5+10
L51	Yi Zhi Mai	N/7+8/2+12
L52	Da Yu Hua	N/7/2+12
L53	Bai Nong 3217	1/7+8/2+12
L54	Xiao San Yue Huang	N/7+8/2+12
L55	Leng Tiao Hong Mai	N/7+8/2+12
L56	Shi Jia Zhuang407	N/7/2+12
L57	Yang Mai 158	N/7+8/2+12
L58	Feng Mai 11	N/7+8/2+12
L59	Bu Jia Mai 7859	N/7+9/2+12
L60	Gan Mai 8	1/7+8/2+12
L61	baipu	N/7+8/2+12
L62	Bai Ma zhaD5703	1/7+8/2+12
L63	Ai Feng 3	1/7+9/2+12
L64	Zi Sui Hong	1/7+8/2+12
L65	Chun Mai D5697	N/7+8/2+12
L66	Ma Li Ying 5	N/7+8/2+12
L67	Xiao Chun 28	N/7+8/2+10
L68	Zhu Shi Mai	N/7+8/2+12
L69	Hui Ning 10	1/7+8/2+12
L70	Pao Zi Mai	N/7+8/2+12
L71	Chi Ke	N/7+8/2+12
L72	Bai Mang Mai	N/7+8/2+12
L73	Song Rui Mai 4	N/7+8/2+12
L74	Shui Yuan 86	N/7+9/2+12
L75	Cang Dong 4	N/7+8/2+12
L76	Shan Mai	N/7+8/2+12
L77	KaBKa3	1/7+8/5+10
L78	Cai Zhou 953	N/7+9/2+12
L79	Gao Yuan 506	N/7+9/2+12
L80	Xiao Yan 506	1/7+8/5+10
L81	D5755	N/7+8/2+12
L82	Da Kou Mai	N/7+8/2+12
L83	Shang Lin Xiao Mai	N/7+8/2+12
L84	Shi Mu-14	N/7+8/2+12
L85	dian xi hong ke	1/13+16/2+12
L86	Da Bai Mai	N/7+8/5+10
L87	D5698	N/7+8/2+12
L88	Guang Feng 3	N/7+8/2+12
L89	Xian Nong 39	1/7+8/5+10
L90	Bai Mang Mai	1/7+8/2+12

L91	Jin Huang Mai	N/7+8/2+12
L92	Mu Jia Dan Ga	N/22/2+12
L93	Qing Chun 28	N/7+8/2+12
L94	Yan Mai 15	1/7+9/2+12
L95	Bai Da Tou	N/7+8/2+12
L96	Fu Mai	1/7+8/2+12
L97	Bai Mang Xiao Mai	N/7+9/2+12
L98	Xin Ke Han 9	1/7+9/2+12
L99	Qiang Chang Mai	N/7+22/2+12
L100	Ping Yin-27	N/7+8/2+12
L101	Tong Jia Ba Xiao Mai	N/7+8/2+12
L102	Yu qiu Mai	N/7+8/2+12
L103	Huang Hua Mai	N/7+8/2+12
L104	D5727 Gan Mai 8	1/7+8/2+12
L105	Ji Chun 1016	2*/7+9/5+10
L106	Loorin-10	2*/7+8/2+12
L107	Jin Mai 2148	N/7+8/2+12
L108	Da Qing Mang	N/7+8/2+12
L109	Hong Mang Mai	N/7+8/2+12
L110	Kang Ding Xiao Mai	N/7+8/2+12
L111	Tanori	1/7+8/5+10
L112	Ao De Sa 3	N/6+8/2+12
L113	Qian Jiao Mai	2*/7+9/2+12
L114	orofer Ou Guo	N/7+8/2+12
L115	Funo A Fu	N/7+8/2+12
L116	Fan 6	N/7+8/2+12
L117	Ri Ka Ze-8	1/7+8/2+12
L118	Bi Ma 1	N/7+8/2+12
L119	Hong Xu Mai	N/7+8/2+12
L120	Hong Hua Mai	N/7+8/2+12
L121	Bian Ba Chun Mai-6	N/7+8/2+12
L122	D5683	N/7+8/2+12
L123	Jiang Chun Mai	N/7+8/2+12
L124	Zheng Zhou 6	1/7+8/2+12
L125	Fu Zhuang 30	1/7+8/2+12
L126	ningchub4	1/7+8/2+12
L127	Hong Hua Zi	1/7+8/2+12
L128	He Shang Mai D655	N/7+8/2+12
L129	Zi Pi	N/7+8/2+12
L130	You Mang Sao Gu Xing	N/7+8/2+12
L131	Cheng Du Guang Tou	N/7+8/2+12
L132	Jin Mai2148	N/7+8/2+12
L133	Han Zhong Bai	N/7+8/2+12
L134	Zhu Gou Mai	N/7+8/2+12
L135	D5695	N/7+8/2+12
L136	Bian Guang Tou Ke Mai	2*/7+9/5+10
L137	Di Xiu Han	N/7+8/2+12

L138	Wu Mang Chun Mai	N/7+8/5+10
L139	Gui Nong 10	N/7+9/2+12
L140	Ban Jie Mang	N/7*+8/2+10
L141	Bai Dong Mai	N/7+8/2+12
L142	Huo Han Yan	N/7+8/2+12
L143	Gao Yuan 506	N/7+9/2+12
L144	Hong Tu Zi	N/7+8/2+10
L145	Shi Jia Zhuang 54	N/7+8/2+12
L146	Han Yang Mai	N/7+8/2+12
L147	Zha Hong	N/7+8/2+12
L148	Jiang Mai	1/7+8/5+10
L149	Hu Bu Hong	N/6+8/5+10
L150	San Yue Huang	1/7+8/2+12
L151	Xiao Yan 6	1/13+16/5+10
L152	Ji Nan 2	N/7+8/2+12
L153	Yang Mai	N/7+8/2+12
L154	Ben Di Huang Hua Mai	N/7+9/2+12
L155	Nei Xiang	N/7+8/2+12
L156	Hong He Shang Tou	N/7+8/2+12
L157	Fu Mai	N/7+8/5+10
L158	C An Xiao Mai	N/7+8/2+12
L159	Chang Mang Shi Bian Tou	N/7+8/2+12
L160	Yun Mai 34	N/7+9/2+12
L161	Huo Mai	N/7+8/2+12
L162	Bai Mang Mai	N/7+8/2+12
L163	Shen Gen	1/7+8/2+12
L164	Bi Mai 26	N/7+8/5+10
L165	Fu Yang Mai	N/7+8/4+12
L166	Xin Shu Guang 6	1/7+8/5+10
L167	D5696	2*/7+8/5+10
L168	Mang Xiao Mai	N/7+8/2+12
L169	Dun Hua Chun Mai	N/7+8/2+12
L170	Bi Ma 4	N/7+8/4+12
L171	Jin Mai 4	1/7+8/2+12
L172	Yang Mai	1/7/4+12
L173	Mo Tuo Xiao Mai	N/7+8/2+12
L174	Yang Mai	N/7+8/2+12
L175	Hong Gou Dou	N/7+8/2+12
L176	Tai Shan -1	N/7+8/5+10
L177	Hua Dong 6	2*/7+9/5+10
L178	Sheng He Xiao Mai	N/7+8/2+12
L179	Che Zi	N/7+8/2+12
L180	Zheng Yin 4	1/13+19/2+12
L181	WA6E142	N/7+9/5+10
L182	You 1083	N/7+9/2+12
L183	Ping Yuan 50	N/7+8/2+12
L184	Zheng Zhou 741	1/7/5+10

L185	Xi Nong 07K1310	N/7+9/2+12
L186	Bai You Mai	N/7+9/2+12
L187	Bai Tu Zi Tou	1/7+8/4+12
L188	Lao Qi Mai	1/7+8/2+12
L189	Chu Shan Bao	N/7+8/2+12
L190	pinⅡ-2	N/7+8/2+12
L191	WA9D4	N/7/2+12
L192	Pin 9	N/7/2+10
L193	WA8E81	1/7+9/2+12
L194	Lan Xi Zao Xiao Mai	N/7+8/2+12
L195	you Bao	1/7+9/5+12
L196	Pin 3	2*/7+8/5+10
L197	Xi Shan Bian Sui	N/7+8/2+10
L198	San Ke Cun	N/13+16/2+12
L199	Xiao Fo Shou	N/7+8/2+12
L200	Xin 27	N/7+8/2+12
L201	Hua 1223	1/7+8/2+12
L202	PJ-2	N/7+8/2+12
L203	WA40167	1/7+9/5+10
L204	DH16	N/7+8/2+12
L205	Fan-2	1/7+8/5+10
L206	Da Bai Mai	N/13+16/2+10
L207	Wu Jiang Cao	N/7+16/2+10
L208	San Yue Huang	N/7+8/2+12
L209	Chong Yang Hong Mai	N/7+8/2+12
L210	Da Tou Chun	N/7+8/2+12
L211	Hong Mai	N/7+8/2+12
L212	Zhu Ye Qing	N/7+8/2+12
L213	Zi Mai	N/7+8/2+12
L214	Yang Xin Da Tou	N/7+8/2+12
L215	Zhu You Bai	N/7+8/2+12
L216	Xun Xi Er Chi Ban	1/7+8/2+12
L217	Xun Xi Hong Ke Mai	N/7+8/2+12
L218	Cao Xie Ban	N/7+8/2+12
L219	Cao Xie Ban	N/7+8/2+12
L220	Li Chuan San Yue Huang-3	N/7+8/2+12
L221	Yi Xi Shi	1/7+8/2+12
L222	Yi Li Piao	N/7+8/2+12
L223	Pu Shan Ba Xiao Mai	N/17+18/2+12
L224	Ba Di Tuo	N/7+9/2+12
L225	Ba Lian Tuo	N/14+15/2+12
L226	Ba Gu Tao	N/7+8/2+12
L227	Ba Gu Tao-2	N/7+8/2+12
L228	San Yue Huang	N/7+8/2+12
L229	San Yue Huang	N/7+9/2+12
L230	San Yue Huang	1/6+8/5+10
L231	San Yue Huang	N/7+8/2+12

L232	San Yue Huang	N/7+8/2+12
L233	San Yue Huang	N/7+8/2+12
L234	San Yue Huang	N/7+8/2+12
L235	San Yue Huang	1/6+8/5+10
L236	San Yue Huang	N/7+8/2+12
L237	San Yue Huang 1	N/7*+8/2+12
L238	San Yue Huang 2	N/7+8/2+12
L239	San Yue Liu Zao Mai	1/7+8/5+10
L240	San Yue Liu Zao Mai	1/7+8/2+12
L241	San Li Yi Cun	N/17+18/N
L242	San Ke Cun	N/7/2+12
L243	Da Bai Mang	N/7+8/2+12
L244	Da Tou Mai	1/7+8/2+12
L245	Da Tou Chun	N/7+8/5+10
L246	Da Tou Huang	N/6+8/5+10
L247	Da Tou Huang	N/7+8/2+12
L248	Da Hong Mang	N/7+8/2+12
L249	Da Hong Mang Mai	N/7+8/2+12
L250	Da Pi Gu Xiao Mai	1/7+8/2+12
L251	Da Sui Mai	1/7+8/2+12
L252	Xiao Duo Bai	N/7+8/2+12
L253	Xiao Hong Mang Mai	N/7+8/5+10
L254	Xiao He Shang Tou	N/7+8/2+12
L255	Xiao He Shang Tou	N/7+8/2+12
L256	Xiao Yang Mai	N/7+8/2+12
L257	Shan Lin Guo Mai	2*/7+9/2+12
L258	Shan Xi Zao	N/7+8/2+12
L259	Qian Jin Bu Dao	N/7+8/2+12
L260	Qian Jin Bu Dao	N/7+8/2+12
L261	Qian Jin Bu Dao	1/7+8/2+12
L262	Qian Jin Bu Dao Fu	N/7+8/2+12
L263	Qian Jin Ding	1/7+9/2+12
L264	Zi Mai	N/7+8/2+12
L265	Yun Nan Zao	N/7*+8/2+12
L266	Wu Yue Zao	N/7+8/2+12
L267	Wu Hong Cao	N/7+8/2+12
L268	Wu Hong Cao	N/7+8/2+12
L269	Wu Hong Cao	1/7+8/2+12
L270	Wu Hong Cao	N/7+8/2+12
L271	Shui Bian Zhi	N/7+8/2+12
L272	Mao Gan	1/7/5+10
L273	Chang Sha Mai	N/7+8/2+12
L274	Chang Xu Mai	N/7+8/2+12
L275	Jin Da Dan	N/7+8/2+12
L276	Liu Yue Huang	N/14+15/2+12
L277	Liu Jin Mu	N/7+8/2+12
L278	Huo Xu Mai	1/7+8/2+12

L279	Huo S Tou	N/7+8/2+12
L280	Huo S Tou	N/7+8/2+12
L281	Huo Yan Mai	N/7+8/2+12
L282	Huo Liao Tou	N/7+8/2+12
L283	Huo Liao Yan	N/7+8/2+12
L284	Huo Wu Tou	1/7+8/2+12
L285	Huo Mai	N/7+9/2+12
L286	Huo Mai	N/7*+8/2+12
L287	Huo Mai	N/7+8/2+12
L288	Huo Mai	N/7+8/2+12
L289	Jin Da Dan	N/7+22/N
L290	Jin Da Dan	1/7+22/2+12
L291	Wu Jin Xiao Mai	N/7+8/2+12
L292	Yu Mai	N/7+22/N
L293	Yu Mai	N/7+9/2+12
L294	Yu Mai	1/7+9/2+12
L295	Bian Zhong Xiao Mai	1/7+8/2+12
L296	Ben Di Xiao Mai	N/13+16/2+12
L297	Ben Di Xiao Mai	1/7+8/2+12
L298	Shi Xia Yang Mai	N/7+8/2+12
L299	Shi Hui Tiao	N/7+9/2+12
L300	Si Fang Luo Xiao Mai	N/7+9/2+12
L301	Bai Xiao Mai	1/7+9/2+12
L302	Bai Ma Ya Xiao Mai	1/13+16/2+12
L303	Bai Huo Deng Tou	N/7+8/2+12
L304	Bai Yu Pi	1/7+8/2+12
L305	Bai Yu Mai	1/7+8/2+12
L306	Bai Pi Mai	N/7+8/2+12
L307	Bai Mang Da Zi	N/7+8/2+12
L308	Bai Mang Xiao Mai	N/7+9/2+12
L309	Bai Mang Guang Tou Mai	N/7+8/2+12
L310	Bai Mang Guang Tou Mai	N/7+8/2+12
L311	Bai Mang Mai	N/7+8/2+12
L312	Bai Mang Mai	1/7+8/2+12
L313	Bai Mang Mai	N/14+15/2+12
L314	Bai Guang Tou 1	1/7+8/2+12
L315	Bai Guang Tou 2	N/7+8/2+12
L316	Bai Guang Tou Mai	1/7+8/2+12
L317	Bai Mai	1/7/2+12
L318	Bai Mai	N/7+8/2+12
L319	Bai Mai	N/7+22/2+12
L320	Bai Mai	1/7+9/2+12
L321	Bai Mai Zi	N/7*+8/2+12
L322	Bai Mai Zi	N/7+8/2+12
L323	Bai Mai Zi	N/7+8/2+12
L324	Bai Ke Jiang	N/7+8/2+12
L325	Bai He Shang Tou	N/7+8/2+12

L326	Bai He Shang Tou	N/7+8/2+12
L327	Bai Ge Ta	N/7+8/2+12
L328	Bai Xu	N/7+22/2+12
L329	Bai Yang La Zi	N/7+8/2+12
L330	Bai Quan Mang	N/7*+8/2+12
L331	Bai Quan Mang	2*/7*+8/2+12
L332	Bai Juan Mang	N/7+8/2+12
L333	Cong Qing Mai	N/7+8/2+12
L334	Cong Qiu Mai	N/7+8/2+12
L335	Ling Xuan 5	1/7+9/2+12
L336	Dong Mai	N/7+8/2+12
L337	Ban Yue Zao	N/7+8/2+12
L338	Tai Xiao Mai	1/7+8/2+12
L339	Ni Qiu Chuan	N/7+8/2+12
L340	Lai Feng Hong Ke Jiang	N/7+8/2+12
L341	Lao Mang Mai	N/7+8/2+12
L342	Lao Hong Xu Mai	N/7+9/2+12
L343	Lao Tie Gan Cao	N/7+8/2+12
L344	Lao Tie Gan Cao	N/14+15/2+12
L345	Mang Zi Mai	N/7+8/2+12
L346	Xi Yang Zao	N/7+8/2+12
L347	Zao Feng Shou	1/7+8/2+12
L348	Guang Tou Xiao Mai	N/20/2+12
L349	Guang Tou Bai Mai Zi	1/7+8/2+12
L350	Guang Guang Tou	N/7+8/2+12
L351	Guang Tu Mai	1/7+8/2+12
L352	Tuan Yu Dan	N/7+8/2+12
L353	Zhu Ye Qing	N/7+8/2+12
L354	Zhu Gan Qing	N/7+8/2+12
L355	Zhu Gan Qing	N/7+8/2+12
L356	Zi Jing Bai	N/7+8/2+12
L357	Qi Tou Huang	N/7+8/2+12
L358	Qi Tou Huang	N/7+8/2+12
L359	Guan Yu Hong	N/7+8/2+12
L360	Hong San Ai	N/7+9/2+12
L361	Hong Xiao Mai	N/7+8/2+12
L362	Hong Mao Qiu	1/7+8/2+12
L363	Hong Mang	1/7+8/2+12
L364	Hong Mang	N/13+16/2+12
L365	Hong Mang	N/7+8/2+12
L366	Hong Mang Zi	N/7+9/2+12
L367	Hong Mang Mai	1/7+8/2+12
L368	Hong Mai	N/7+8/2+12
L369	Hong Ke Chong	2*/7+8/2+12
L370	Hong Ke Mai	N/7+9/2+12
L371	Hong Ke Mai	N/7+8/2+12
L372	Hong Hua Zao	N/7+8/2+12

L373	Hong He Shang Tou	N/7+8/5+10
L374	Hong He Shang Tou	2*/7+8/5+10
L375	Hong He Shang Tou	N/7+8/2+12
L376	Hong He Shang Tou	N/7+8/2+12
L377	Hong He Shang Tou	N/7+8/2+12
L378	Hong Xu	N/7+8/2+12
L379	Hong Xu Mai	2*/7+8/2+12
L380	Hong Xu Mai	N/7+8/2+12
L381	Hong Quan Mang	N/7+8/2+12
L382	Hong Quan Mang	N/7+8/2+12
L383	Hong Quan Mang	N/7+8/2+12
L384	Hong Tao Mai	N/7+8/2+12
L385	Hong Tao Mai	2*/7+8/2+12
L386	Hong Mang	N/7+8/2+12
L387	Guan Yin Lian	N/7+8/2+12
L388	Mai Er Dong Xiao Mai	N/7+8/4+12
L389	Xiao Gan Mai	1/20/2+12
L390	Dou Mai	1/20/2+12
L391	Ying Shan Da Gu Chui	N/7+8/2+12
L392	Leng Sha Cao	N/7+8/2+12
L393	Qing Yi	N/13+16/2+12
L394	Feng Shu Qiu	2*/7+8/2+12
L395	Feng Ye Qiu	2*/7+8/2+12
L396	Yu Lin Zi 1	N/7+8/2+12
L397	Yu Lin Zi 2	N/7+8/2+12
L398	Yu Lin Zi 3	2*/7+8/2+12
L399	He Shang Tou	N/7+8/2+12
L400	He Shang Tou	N/14+15/2+12
L401	He Shang Tou	2*/7+8/2+12
L402	He Shang Tou	N/7+8/2+12
L403	He Shang Tou	1/7+9/2+12
L404	He Shang Tou	2*/7+8/2+12
L405	He Shang Tou 1	1/7+9/2+12
L406	He Shang Tou 2	N/20/2+12
L407	He Shang Mai	N/7+8/2+12
L408	He Shang Mai	N/17+18/2+12
L409	He Shang Mai	N/14+15/2+12
L410	Jin Da	N/7+8/2+12
L411	Jin Guang Mai	2*/7+8/2+12
L412	Yu Lin Bai	N/7+8/2+12
L413	Mei Yu Mai	2*/13+16/5+10
L414	Mei Guo Yu	N/7+8/2+12
L415	He Nan Cao	N/7+8/2+12
L416	He Nan Cao	2*/7*+8/2+12
L417	Pao Mai	N/7+9/2+12
L418	Ni Mai	N/20/2+12
L419	Yi Du Xiao Mai	2*/7+8/2+12

L420	Shi Gan	N/7+9/5+10
L421	Nan Jing Mei	N/7/2+12
L422	Nan Sang Zi	N/14+15/2+12
L423	Cao Xie Bian	N/7+9/5+10
L424	Cao Xie Bian Xiao Mai	2*/7+8/2+12
L425	Cao Xie Ban	N/7+8/2+12
L426	Cao Xie Ban	2*/7+8/2+12
L427	Huang Zi Mai	N/7+8/2+12
L428	Dian Tou Mai	N/7+8/2+12
L429	Pei Shan Mai	N/7*+8/2+12
L430	Sheng Li Hong	N/7+8/2+12
L431	Z Mai	N/7+8/2+12
L432	Ma Yi Dan	2*/7+8/2+12
L433	Xun Xi Dao Qi	N/7*+8/2+12
L434	Ha Dan San	N/7+8/2+12
L435	Bai Mao	N/7+8/2+12
L436	Zhong Qing Mai	N/7+8/2+12
L437	Liu Mang Mai	N/7+8/2+12
L438	Zi Mai	N/7+8/2+12
L439	Zi Mai	N/7+8/2+12
L440	Yang Mai	N/7+8/2+12
L441	Yang Mai	2*/7+8/2+12
L442	Yang La Zi Xiao Mai	N/7+8/2+12
L443	San Dan	N/7+8/2+12
L444	Dun Ban	N/20/2+12
L445	Yuan Du Mai	N/7+8/2+12
L446	Yuan Zhui	2*/7+8/2+12
L447	Can Dou Mai	N/7*+8/2+12
L448	Tie Zi Mai	N/7+8/2+12
L449	Tie Gan Ai	1/6+8/2+12
L450	Tie Gan Cao	1/7+8/2+12
L451	Tie Gan Cao	N/7+8/2+12
L452	Tie Gan Cao	N/7+8/2+12
L453	Tie Gan Cao 1	N/7+8/2+12
L454	Tie Gan Cao 2	N/7+8/2+12
L455	Tie Zi Mai	N/13+16/2+12
L456	Tie Zi Mai	N/7+8/2+12
L457	Gao Shan Xiao Mai	N/7+8/2+12
L458	Quan Mang	N/7+8/2+12
L459	Quan Mang	N/7+8/2+12
L460	Quan Mang	N/7+8/2+12
L461	Quan Mang Xiao Mai	N/7+8/2+12
L462	Quan Mang Mai	N/14+15/2+12
L463	Shui Ba Gu Tao	N/7+8/2+12
L464	Shan Zi Mai	N/7+8/2+12
L465	Qiu Mai	N/7*+8/2+12
L466	Huang Shu Lang Pi	N/7+8/2+12

L467	Cai Zi Huang Xiao Mai	N/7+8/2+12
L468	Zi Tou	N/7+8/2+12
L469	E Yu Zao	N/7+8/2+12
L470	Chong Yang Xiao Mai 1	N/7+8/2+12
L471	Chong Yang Xiao Mai 2	N/20/2+12
L472	Chong Yang Hong Mai 1	N/7+8/2+12
L473	Chong Yang Hong Mai 2	N/7+8/2+12
L474	Li Dao Mai	N/7+8/2+12
L475	Zi Mai	N/7+8/2+12
L476	Zi Mai	N/7+8/2+12
L477	Qin Ke Mai	N/7+9/2+12
L478	Xi Ma Zi Mai	N/7+9/2+12
L479	Wo Jiao Mai	N/13+16/2+12
L480	Pu Shan Ba	N/7+8/2+12
L481	Pu Shan Ba	N/7+8/2+12
L482	Pu Shan Ba	N/7+8/2+12
L483	wu gong Chong 1	N/7+8/2+12
L484	wu gong Chong 2	N/14+15/2+12
L485	wu gong mai	N/7+8/2+12
L486	wu gong mai	N/7+8/2+12
L487	wu gong mai	N/7+8/2+12
L488	wu gong mai	N/7+8/2+12
L489	wu gong mai 3	2*/7+8/2+12
L490	wu gong Xu	N/7*+8/2+12
L491	wu gong Xu	N/7+8/2+12
L492	wu gong Cao	N/7*+8/2+12
L493	wu gong Cao	N/7+8/2+12
L494	wu gong Cao	N/7+8/2+12
L495	Chui Zi Ba	N/7+8/2+12
L496	Ai Jiao Huang	N/7+8/2+12
L497	Huai Shu Qiu	N/7+8/2+12
L498	Duan Yang Zao	N/7+8/2+12
L499	Luo Zi Mai	N/7+8/2+12
L500	Luo Si Mai	N/7+8/2+12
L501	Luo Si Mai	N/7+8/2+12
L502	Heng Zi Mai	N/7+8/2+12
L503	Kao Shan Hong	N/7+8/2+12
L504	ji Yu Bao	N/7+8/2+12
L505	ji Yu Bao	N/7+8/2+12
L506	ji Yu Bao	N/7+8/2+12
L507	ji Yu Bao	N/7+8/2+12
L508	ji Yu Zi	N/7+8/2+12
L509	ji Yu Po	N/7+8/2+12
L510	ji Yu Bao	2*/7+8/2+12
L511	Bao Di Jia	N/7+8/2+12
L512	Bao Di Jiang	N/7+8/2+12
L513	Bao Di jiang	N/7+8/2+12

L514	Yi Gen Dan	N/7*+8/2+12
L515	Yi Ke Miao	N/7+8/2+12
L516	San Yue Huang	N/7+9/2+12
L517	San Yue Huang	N/7+8/2+12
L518	San Yue Huang	N/7+8/2+12
L519	San Yue Huang	N/7+8/2+12
L520	San Yue Huang	N/7+8/2+12
L521	San Yue Huang	N/7+8/2+12
L522	San Yue Huang	N/7+8/2+12
L523	San Li Cun	N/13+16/2+12
L524	San Ke Cun	N/13+16/2+12
L525	San Ke Cun	N/13+16/2+12
L526	Da Hong Mang	N/7+8/2+12
L527	Da Mai Zi	N/7+8/2+12
L528	Da He Shang Tou	N/7+9/2+12
L529	Xiao Xiao Mai	N/7+8/2+12
L530	Xiao Mai	N/7+8/2+12
L531	Xiao Mai	N/7+8/2+12
L532	Xiao Tie Zi Mai	N/13+16/2+12
L533	Liu Leng Zi	N/7+8/2+12
L534	Ben Di Mai	N/7+8/2+12
L535	Bai Xiao Mai	N/7+8/2+12
L536	Bai Xiao Mai	N/7+8/2+12
L537	Bai Xiao Mai	N/7+8/2+12
L538	Bai Mai	1/7+9/2+12
L539	Bai Mai	N/7+9/2+12
L540	Bai Mai Zi	N/13+16/2+12
L541	Bai Mai Zi	N/7+8/2+12
L542	Bai Mai Zi	N/7+8/2+12
L543	Bai Ke Jiang	N/7+8/2+12
L544	Bai He Shang Tou	N/7+8/2+12
L545	Lao Xiao Mai	N/7+8/2+12
L546	Lao Mai Zi	N/7+22/2+12
L547	Guang Tou Mai	N/7+8/2+12
L548	Quan Mang Zi	N/7+8/2+12
L549	Quan Mang Zi	N/7+8/2+12
L550	Hong Xiao Mai	N/7+8/2+12
L551	Hong Pi Mai	N/7+8/2+12
L552	Hong Mang Zi	N/7+8/2+12
L553	Hong Mang Zi	2*/7+8/2+12
L554	Hong Mang Mai	N/7+8/2+12
L555	Hong Mang Mai	N/7+8/2+12
L556	Hong Mang Mai	N/7+8/2+12
L557	Hong Mang Mai	N/7+8/2+12
L558	Hong Mang Mai	N/7+8/2+12
L559	Hong Mang Mai	N/7*+8/2+12
L560	Hong Mang Li Jian Mai	N/7+8/2+12

L561	Hong Mai	N/7+8/2+12
L562	Hong Mai	N/7+8/2+12
L563	Hong Mai	N/7+8/2+12
L564	Hong Hua Xiao Mai	N/7+8/2+12
L565	Hong Xu Mai	N/7+8/2+12
L566	Su Ke Xi	N/7*+8/2+12
L567	He Shang Tou	N/7+8/5+10
L568	He Shang Tou	1/7+8/2+12
L569	He Shang Tou	2*/7+8/2+12
L570	Shan Xi Xiao Mai	2*/7+8/2+12
L571	Shan Xi Zao	N/7+8/2+12
L572	S Mai	N/13+16/2+12
L573	Cao Zi Huang	N/7+9/2+12
L574	Cao Xie Ban	N/7+8/2+12
L575	Cao Xie Ban	N/7+9/2+12
L576	Nan Da Bai Mai	2*/7+8/2+12
L577	Nan Da Mai	N/7+8/2+12
L578	Nan Xiao Mai	N/7+8/2+12
L579	Nan Shan Xiao Mai	2*/7+8/2+12
L580	Nan Jing Zao	N/7+8/2+12
L581	Nan Jing Zi	N/20/2+12
L582	Nan Jing Zi	N/7+8/2+12
L583	Ha Zi Wu Mai	N/7+9/2+12
L584	Yang Xiao Mai	N/7+8/2+12
L585	Tie Gan Cao	N/7+8/2+12
L586	Tie Gan Cao	N/7+8/2+12
L587	Tie Gan Cao	N/7+8/2+12
L588	Gao Gan Xiao Mai	1/7+8/2+12
L589	Quan Wang Mai	N/7+8/2+12
L590	Pai Deng Mai	N/7+8/2+12
L591	Ben Mai	N/7+8/2+12
L592	Duan Gan Bai	N/7+8/2+12
L593	Duan Gan Duo	1/7+9/2+12
L594	Ai Gan Hong	2*/7+9/2+12
L595	Ai Gan Hong	N/7+8/2+12
L596	Ke Li Bai	N/7+8/2+12
L597	Ke Li Jian	N/7+8/2+12
926 commercial varieties		
504 commercial varieties from HWWR		
C1	Han 5316	1/7+8/2+12
C2	Han 6172	1/14+15/2+12
C3	Han Mai 17	N/7+8/5+10
C4	Han Mai 18	1/13+16/5+12
C5	Hua 521	N/7+9/2+12
C6	Ji 5385	1/7+8/5+10
C7	Ji Mai 15	2*/7+9/2+12
C8	Ji Mai 19	N/7+9/2+12

C9	Ji Mai 20	2*/7+9/2+12
C10	Ji Mai 27	N/7+8/2+12
C11	Ji Mai 325	N/7+9/5+10
C12	Ji Mai 3	N/7+8/2+12
C13	Ji Mai 42	N/7+9/2+12
C14	Ji Mai 7	2*/7+9/2+12
C15	Ji Mai 41	N/7+9/2+12
C16	Ji Mai 86	N/7+8/2+12
C17	Ji Mai 94	1/7+9/2+12
C18	Ji Mai 95	1/17+18/5+10
C19	Ji Mai 96	1/14+15/4+12
C20	Jing Sheng Mai 1	1/7+9/2+12
C21	Shi Mai 22	N/7+9/2+12
C22	Shi Xin 618	N/7+9/2+12
C23	Shi Xin 828	N/7+8/2+12
C24	Yun Han 719	N/7+9/5+10
C25	CA1062	N/7+8/5+10
C26	Bei Jing 8	N/7+8/2+12
C27	Cang Mai 028	N/13+16/2+12
C28	Dong Xie 2	N/7+9/2+12
C29	Feng Kang 2	N/7+9/2+12
C30	Gao You 5766	1/7+8/5+10
C31	Han 4589	N/7+9/2+12
C32	Han Nong 1412	N/7+8/2+12
C33	Han You 3475	2*/7+9/5+10
C34	He Nong 130	2*/7+9/5+10
C35	He Nong 326	N/7+9/2+12
C36	He Nong 58-3	N/7+8/5+10
C37	He Nong 7069	1/7+8/5+10
C38	Heng 4338	N/7+9/2+12
C39	Heng 6599	N/14+15/2+12
C40	Heng S29	N/7+9/2+12
C41	Ji Mai 17	1/7+9/2+12
C42	Ji Mai 26	N/7+9/2+12
C43	Ji Mai 585	1/7+9/2+12
C44	Jie Mai 19	1/7+8/2+12
C45	Jin Feng 12-449	1/7+9/2+12
C46	Jin He 12-088	1/7+9/2+12
C47	Jin He 12-089	1/7+9/2+12
C48	Jin He 12-339	1/7+9/2+12
C49	Jin He 7178	1/7+8/2+12
C50	Jin He 8211	1/7+9/2+12
C51	Jin Tai 9923	N/7+9/2+12
C52	Jing Ai 21	N/7+9/2+12
C53	Jing Dong 18	N/7+8/2+12
C54	Jing Dong 23	N/7+8/2+12
C55	Jing Hua 11	N/7+9/2+12

C56	Jing Nong 79-13	N/7+9/2+12
C57	Nong Da 212	N/6+8/2+12
C58	Nong Da 399	1/7+9/2+12
C59	Nong Da 408	N/7+9/2+12
C60	Nong Da 4123	N/7+9/2+12
C61	Nong Da 5363	N/7+9/5+10
C62	Ping Yang 56	2*/7+8/5+12
C63	Shi Mai 14	1/7+9/2+12
C64	Shi Mai 18	N/7+9/2+12
C65	Shi Nong 086	N/7+8/5+10
C66	Tai 5902	N/7+8/2+12
C67	Ying Bo 700	N/7+8/5+10
C68	Yong Mai 3	N/7+8/2+12
C69	Zhong Mai 113	N/7+9/2+12
C70	Zhong Mai 1197	N/7+9/2+12
C71	Zhong Mai 533	1/7+9/2+12
C72	Han Mai 9	1/14+15/2+12
C73	He Nong 215	N/7+9/2+12
C74	Ji Mai 23	1/7+9/2+12
C75	Ji Mai 24	N/7+9/2+12
C76	Jing Zuo 278	N/7+9/2+12
C77	Bei Jing 8694	N/7+9/2+12
C78	Bei Nong 2	N/7+9/2+12
C79	Cang 6003	N/7*+8/2+12
C80	Cang Mai 12	1/7+9/5+10
C81	Cang Mai 6001	N/7+8/2+12
C82	Cang Mai 6005	N/7*+8/2+12
C83	Feng Kang 13	N/6+8/2+12
C84	Han 05-5092	1/14+15/2+12
C85	Han Dan 6050	1/7+9/2+12
C86	Han Mai 16	1/14+15/2+12
C87	Han Xuan 11	N/7+8/5+10
C88	Han Xuan 12	N/7+9/2+12
C89	Han Xuan 2	N/7+8/5+10
C90	He Nong 6049	N/7+9/2+12
C91	He Nong 7106	1/7+9/2+12
C92	He Nong 827	1/14+15/2+12
C93	He Nong 85-9	N/7+9/2+12
C94	Heng 0816	N/7+9/2+12
C95	Heng 08 Guan 29	N/7+9/2+12
C96	Heng 136	N/7+9/2+12
C97	Heng 4041	N/7+9/2+12
C98	Heng 4399	1/14+15/2+12
C99	Heng 4444	1/14+15/2+12
C100	Heng 5229	1/7+9/2+12
C101	Heng 6632	1/7+9/2+12
C102	Heng 7228	2*/7+9/5+10

C103	Heng 95 Guan 26	1/7+9/2+12
C104	Heng Shui 6404	N/7+8/2+12
C105	Heng You 18	1/7+8/5+10
C106	Hua Bei 187	N/7+9/2+12
C107	Ji 5265	1/7+9/2+12
C108	Ji Feng 703	N/7+9/2+12
C109	Ji Mai 10	N/7+9/2+12
C110	Ji Mai 18	1/7+9/2+12
C111	Ji Mai 29	N/13+16/2+12
C112	Ji Mai 2	N/6+8/2+12
C113	Ji Mai 36	N/7+9/2+12
C114	Ji Mai 38	N/7+8/2+12
C115	Ji Mai 418	N/7+9/5+10
C116	Ji Mai 518	N/7+9/2+12
C117	Ji Mai 6	N/7+9/2+12
C118	Ji Mai 9	N/7+8/2+12
C119	Jian 26	1/7+9/2+12
C120	Jin 2148-7	N/7+8/2+12
C121	Jin Mai 13	N/7+8/2+12
C122	Jin Mai 16	N/7+9/2+12
C123	Jin Mai 17	N/7+8/5+10
C124	Jin Mai 25	N/7+8/5+10
C125	Jin Mai 33	N/7+9/2+12
C126	Jin Mai 39	N/7+8/5+10
C127	Jin Mai 44	N/7+8/2+12
C128	Jin Mai 47	N/7+9/5+10
C129	Jin Mai 50	N/7+8/5+10
C130	Jin Mai 51	N/7+8/2+12
C131	Jin Mai 53	N/7+9/2+12
C132	Jin Mai 54	N/7+9/2+12
C133	Jin Mai 60	N/7+9/2+12
C134	Jin Mai 63	N/7+8/2+12
C135	Jin Mai 68	2*/7+8/5+10
C136	Jin Mai 72	1/7+9/2+12
C137	Jin Mai 79	N/7+9/2+12
C138	Jin Mai 91	N/7+8/2+12
C139	Jin Mai 97	N/7+9/5+10
C140	Jin Mai 97 (Han)	N/7+9/2+12
C141	Jin Tai 102	N/7+9/2+12
C142	Jin Tai 182	N/7+9/2+12
C143	Jing 411	N/7+8/2+12
C144	Jing Dong 8	N/7+9/2+12
C145	Jing He 9822	N/7+8/2+12
C146	Jing Hua 1	N/7+9/5+10
C147	Jing Shuang 16	N/7+9/2+12
C148	Ke Yi 26	N/7+8/2+12
C149	Lin 138	N/7+8/2+12

C150	Lin Y7287	1/7+8/2+12
C151	Lin Fen 8050	1/7+8/5+10
C152	Lin Feng 3	N/7+9/2+12
C153	Lin Feng 615	N/7+9/2+12
C154	Lin Han 5089	N/7+9/2+12
C155	Lin Han 5369	N/7+8/5+10
C156	Lin Han 6105	N/7+8/2+12
C157	Lin Han 917	N/7+9/2+12
C158	Lin Han 935	N/7+9/2+12
C159	Lin Kang 5108	N/7+9/2+12
C160	Lin You 2069	N/7+9/2+12
C161	Lun Kang 7	N/7+9/2+12
C162	Lun Xuan 103	N/7+9/5+10
C163	Nong Da 135	N/7+9/2+12
C164	Nong Da 152	1/7+8/2+12
C165	Nong Da 155	N/7+8/2+12
C166	Nong Da 183	N/7+8/2+12
C167	Nong Da 2427	N/7+9/2+12
C168	Nong Da 311	N/7+9/2+12
C169	Nong Da 3492	N/7+9/2+12
C170	Nong Da 36	1/7+8/5+10
C171	Nong Da 3634	N/7+9/2+12
C172	Nong Da 3659	N/7+9/2+12
C173	Ping Yang 348	N/7+8/2+12
C174	Shi 4185	1/7+9/2+12
C175	Shi Jia Zhuang 407	N/7+9/2+12
C176	Shi Jia Zhuang 8	N/7+9/2+12
C177	Shi Mai 12	1/14+15/5+10
C178	Shi Mai 13	N/7+9/2+12
C179	Shi Mai 15	N/7+9/2+12
C180	Shi Mai 19	1/7+9/5+10
C181	Shi Xin 616	1/7+8/2+12
C182	Shi Xin 633	1/7+8/2+12
C183	Shun Mai 1718	1/14+15/5+10
C184	Tai 13606	N/7+9/2+12
C185	Tai 712	N/7+8/2+12
C186	Tai Yuan 566	N/7+8/2+12
C187	Xiao Shan 8	N/7/2+11
C188	Xiao Yan 54	1/14+15/2+12
C189	Xing Mai 6	1/14+15/2+12
C190	Yan 99102	1/7+8/5+10
C191	Yuan Dong 834	N/7+8/2+12
C192	Yuan Dong 847	N/14+15/2+12
C193	Chang 4640	N/7+9/2+12
C194	Chang 4738	N/6+8/5+10
C195	Chang 4853	N/7+9/2+12
C196	Chang 5259	1/14+15/2+12

C197	Chang 6154	N/7+8/2+12
C198	Chang 6359	N/6+8/5+10
C199	Chang 6452	N/7+8/2+12
C200	Chang 6794	N/7+8/2+12
C201	Chang 6878	1/7+8/5+10
C202	Chang 8744	N/7+9/2+12
C203	Chang Wu 89 (1)3-4	N/7+8/2+12
C204	Chang Zhi 516	N/7+8/2+12
C205	Chang Zhi 620	N/7+8/5+10
C206	Zhong Mai 175	N/7+9/2+12
C207	Zhong Mai 816	N/7+8/2+12
C208	Zhong Mai 996	N/7+8/2+12
C209	Zi You 6	1/7+8/5+10
C210	05F3-244	N/7+9/2+12
C211	05F4-221	N/7+8/5+10
C212	05Z3-155	N/7+8/5+10
C213	06Z5-027	N/14+15/5+10
C214	09F4-252	N/7+9/5+10
C215	09F6-590	1/7+8/5+10
C216	09F6-622	1/7+8/5+10
C217	15A-191	N/7+8/5+10
C218	15 Chan A14	N/7+9/5+10
C219	15 Chan B14	N/7+9/5+10
C220	2010F6-541	1/7+9/2+12
C221	PH82-2-2	1/7/2+12
C222	PH85-16	1/14+15/2+12
C223	Gao You 503	1/7+8/2+12
C224	Gao You 5218	1/7+8/5+10
C225	Gao You 9409	N/7+8/2+12
C226	Gao You 9415	1/7+8/5+10
C227	Gao You 9618	1/7+9/5+10
C228	Han 09-41344	N/7+8/5+10
C229	Han 10-5223	1/7/5+10
C230	Han 7086	1/7+9/2+12
C231	Han Mai 14	N/7+9/2+12
C232	He Nong 185	1/14+15/5+10
C233	He Nong 336	1/7+9/5+10
C234	He Nong 633	1/7+9/5+10
C235	Heng 2011 Guan 272	N/7+9/5+10
C236	Heng S13-5022	1/7+9/5+10
C237	Ji Mai 541	1/7+8/5+10
C238	Ji Mai 738	1/7+8/5+10
C239	Jin Mai U80	1/7+8/5+10
C240	Ji Nuo 200	N/7+8/2.2+12
C241	Jin He 14-383	1/13+16/5+10
C242	Jin Nong 6	1/7+8/5+10
C243	Jing Hua 10	N/7+9/2+12

C244	Ke Nong 2009	1/7+8/5+10
C245	Ke Nong 213	1/7+9/5+10
C246	Nong Da 5181	1/14+15/2+12
C247	Nong Da 6812	N/7+8/2+12
C248	Shi 02-1	N/7+9/2+12
C249	Shi 4045	1/7+8/5+10
C250	Shi U10-4045	1/7+8/5+10
C251	Shi U12-5241	1/7+9/2+12
C252	Shi You 17	1/7+9/5+10
C253	Shi You 20	1/7+8/5+10
C254	Yu Mai 13	N/7+8/5+10
C255	Zhong You 206	1/7+9/5+10
C256	Zhong You 335	N/7+9/2+12
C257	Zhong You 9507	1/7+9/5+10
C258	04SS Fan 04	1/7+8/5+10
C259	04SS Fan 08	1/7+8/5+10
C260	04SS Fan 10	1/14+15/5+10
C261	04 Chu 113	1/6+8/5+10
C262	04 Chu 121	1/14+15/2+12
C263	04 Chu 80	1/7+8/2+12
C264	05Z4-004	N/14+15/5+10
C265	05Z4-020	N/14+15/5+10
C266	05Z4-031	1/14+15/5+10
C267	05Z6-014	1/14+15/5+10
C268	05Z6-369	1/7+8/5+10
C269	05 Hei Chu 27	1/14+15/5+10
C270	05 Hei Chu 8	N/7+8/5+10
C271	06Z6-040	N/7+8/5+10
C272	08F5-287	N/7+9/5+10
C273	08F5-59	N/7+9/5+10
C274	Heng Guan 35	N/7+9/2+12
C275	Ji Xian 3097	N/7+8/2+12
C276	Ji 5579	N/7/2+12
C277	Ji Zi 439	N/14+15/5+10
C278	Jin He 13-294	N/7+8/2+12
C279	Jin Mai 85	N/7+8/2+12
C280	Jin Mai 88	1/14+15/5+10
C281	Jin Mai 89	1/7+8/5+10
C282	Jing Mai 9	N/7+9/2+12
C283	Ke Nong 1002	N/7+9/2+12
C284	Ke Nong 1006	N/7+9/2+12
C285	Ke Nong 199	N/7+9/2+12
C286	Ke Nong 2011	N/7+9/2+12
C287	Ke Nong 9204	N/14+15/2+12
C288	Ke Yi 5214	N/14+15/2+12
C289	Lun Xuan 169	N/7+9/2+12
C290	Nong Da 211	N/6+8/2+12

C291	Nong Da 3432	N/7+8/2+12
C292	Nong Da 413	1/13+16/5+10
C293	Nong Da 5182	N/14+15/2+12
C294	Shun Mai 612	N/14+15/2+12
C295	Tai 10604	N/7+8/5+10
C296	Xing Mai 13	1/14+15/2+12
C297	Yan Da 1817	N/7+8/2+12
C298	Yao Mai 16	N/7+8/2+12
C299	Chang 5222	N/7+8/2+12
C300	Chang Mai 251	N/7+9/2+12
C301	Chang Mai 5973	N/7+9/2+12
C302	Zhong Mai 12	1/7+8/5+10
C303	Zhong Xin Mai 9	1/17+18/5+10
C304	Zi You 5	1/7+8/2+12
C305	Ai Kang 58	1/7+8/5+10
C306	Ai Kang 58-1	1/7+8/5+10
C307	Luo Mai 21	1/7+9/2+12
C308	Luo Mai 6010	1/7+9/5+10
C309	Ping An 7	N/7+8/2+12
C310	Ping An 8	1/7+9/2+12
C311	Shen Mai 1	1/7+8/5+10
C312	Xin Mai 29	N/14+15/2+12
C313	Xin Mai 30	1/7+9/2+12
C314	Yan Gao 21	N/7+9/2+12
C315	Yu Mai 18	N/13+16/5+10
C316	Zheng Yu Mai 9989	N/7+9/5+10
C317	Zhou Mai 22	1/7+9/2+12
C318	Zhou Mai 25	1/7+9/2+12
C319	Zhou Mai 32	1/7+9/5+10
C320	Zhou Mai 37	1/7+9/5+10
C321	Zhou Mai 38	1/7+9/5+10
C322	Zhou Mai 39	N/7+8/5+10
C323	Zhou Mai 40	1/7+9/5+10
C324	Jun Mai 99-7	1/7+9/5+10
C325	Kai Mai 21	1/7/2+12
C326	Lan Kao Ai Zao 8	N/7+9/2+12
C327	Nei Nong Ke 201	1/7+9/5+10
C328	Tian Min 198	1/14+15/5+10
C329	Wen 9629	1/7+9/2+12
C330	Xin Mai 208	N/14+15/5+10
C331	Xu Ke 718	1/7+9/2+12
C332	Zhan 4110	N/14+15/5+10
C333	Yu Jiao 5	1/7+9/5+10
C334	Yu Mai 158	1/7+9/5+10
C335	Yu Mai 16	N/7+9/2+12
C336	Yu Mai 18	N/14+15/5+10
C337	Yu Mai 34	1/7+8/5+10

C338	Yu Mai 416	1/7+8/5+10
C339	Zheng Feng 9962	N/7+8/5+10
C340	Zheng Mai 101	N/7+8/2+12
C341	Zhong Jiao 2	N/7+8/2+12
C342	Zhong Luo 08-1	1/7+8/2+12
C343	Zhong Mai 998	N/7+8/2+12
C344	Zhou Hei Mai 1	N/7+8/2+12
C345	Zhou Mai 16	N/7+9/2+12
C346	Zhou Mai 28	1/7+9/5+10
C347	Zhou Mai 30	1/7+9/2+12
C348	An Mai 1	1/14+15/5+10
C349	Guo Mai 301	1/14+15/5+10
C350	He Mai 2	N/7+8/2+12
C351	Jun-16	N/7+9/2+12
C352	Jun Xiao 9706	1/7+9/2+12
C353	Luo Han 10	N/7+9/2+12
C354	Luo Han 11	1/7+8/2+12
C355	Luo Han 13	N/7+9/2+12
C356	Luo Han 2	1/7+8/2+12
C357	Luo Han 3	1/7+9/2+12
C358	Luo Han 6	N/7+8/2+12
C359	Luo Han 7	1/7+8/2+12
C360	Luo Mai 22	1/7+9/2+12
C361	Luo Mai 24	N/7+8/5+10
C362	Luo Mai 26	1/7+9/5+10
C363	Luo Yang 8628	N/7+8/2+12
C364	Luo Mai 18	1/7+9/2+12
C365	Luo Mai 7	1/7+9/2+12
C366	Luo You 7	N/7+9/5+10
C367	Xin Mai 20	N/7+8/2+12
C368	Xu Nong 5	1/7+9/2+12
C369	Yi Mai 6	1/7+9/2+12
C370	Yu Jiao Hei 1	2*/7+9/5+10
C371	Yu Mai 29	N/7+8/2+12
C372	Yu Mai 38	1/7+8/2+12
C373	Yu Mai 48	N/7+8/2+12
C374	Yu Mai 50	1/7+9/2+12
C375	Yu Mai 8	1/7+9/2+12
C376	Yu Nong 211	N/13+16/5+10
C377	Yu Nong 949	1/7+8/5+10
C378	Yu Zhan 4	1/7+9/2+12
C379	Zheng Nong 17	1/7+9/5+10
C380	Ji Mai 4	1/13+16/5+10
C381	Xin Mai 18	1/7+9/5+10
C382	Xin Mai 26	1/7+8/5+10
C383	Xin Mai 28	1/7+9/5+10
C384	Xin Mai 1998	1/14+15/5+10

C385	Yan Zhan 1	N/14+15/5+10
C386	Yu Nong 6326	1/7+9/5+10
C387	Zheng Mai 0856	N/7+9/5+10
C388	Zheng Mai 119	1/7+8/5+10
C389	Zheng Mai 1325	N/7+9/5+10
C390	Zheng Mai 366	1/7+8/5+10
C391	Zheng Mai 98	1/14+15/5+10
C392	Zhou Mai 19	1/7+9/5+10
C393	Zhou Mai 24	1/7+9/2+12
C394	Zhou Mai 33	1/7+8/5+10
C395	Zhou Mai 35	1/7+8/5+10
C396	Lan Kao 198	1/13+16/5+10
C397	Yu Mai 13	1/7+8/5+10
C398	Yu Mai 21	2*/7+9/5+10
C399	Yu Mai 982	1/7+9/5+10
C400	Zheng Mai 0943	N/7+9/5+10
C401	Zheng Mai 761	N/7+9/2+12
C402	Zhou Mai 18	1/7+9/2+12
C403	You Zi Tou	N/7+8/2+12
C404	Luo Mai 29	2*/13+16/5+10
C405	Luo Mai 618	N/7+8/5+10
C406	Zheng Mai 2012 H14-23-21	N/7+9/5+10
C407	Zhou Kou 803	1/7+9/5+10
C408	Zhou Kou 608	1/7+9/5+10
C409	Ji Yan Mai 06039	1/7+9/2+12
C410	Luo Mai 31	1/7+9/5+10
C411	Luo Mai 906	1/7+9/2+12
C412	Xin Mai 12037-47-12	N/7+8/5+10
C413	Ji Yan Mai 10	1/7+8/5+10
C414	Zhou 37	1/7+9/5+10
C415	Luo Mai 36	1/7+9/2+12
C416	Luo Mai 903	1/7+8/2+12
C417	Luo Mai 27	1/7+9/5+10
C418	Wan Ke 06229	N/7+8/5+10
C419	Fu Mai 936	1/7+9/5+10
C420	Wan Mai 50	1/7+9/2+12
C421	Ping An 6	N/7+8/2+12
C422	Zhong Tai 1	1/7+9/5+10
C423	Xin Mai 13	1/7+8/5+10
C424	Tai Kong 7	N/7+9/2+12
C425	Dong Feng 1611-1	N/17+18/2+12
C426	Dong Feng 1611-2	N/7+9/2+12
C427	Xi Nong 2208	1/7+8/2+12
C428	Xiao Yan 22	1/7+9/2+12
C429	Yan Mai 8911	N/7+9/2+12
C430	Wan Mai 38	1/7+8/5+12
C431	Yu Mai 20	1/7+8/4+12

C432	Jing Dong 6	N/7+9/2+12
C433	Lun Kang 6	N/7+9/2+12
C434	Jing 437	N/7+8/2+10
C435	Heng Mai 1	1/7+9/2+12
C436	Fu Zhuang 30	N/7+8/2+12
C437	Wan Ke 06230	N/7+8/5+10
C438	Fu Mai 937	1/7+9/5+10
C439	Wan Mai 51	1/7+9/2+12
C440	Ping An 7	N/7+8/2+12
C441	Shan Xi 107	1/7+8/2+12
C442	Lu Mai 1	1/7+8/2+12
C443	Xi Nong 6028	N/7+8/2+10
C444	Fu Mai 368	N/7+8/2+12
C445	Yu Nong 98	1/7+8/2+12
C446	Luo Mai 163	1/7+9/5+12
C447	Lun Xuan 0378	N/7+9/2+12
C448	Shan Nong A064-11	N/7+8/2+12
C449	Shan Nong A52-2	N/7+8/2+12
C450	Shan Nong A079-8	N/7+8/5+12
C451	Shan Nong 076-2	N/7+8/5+12
C452	Shi Mai 22	N/6+8/2+12
C453	Xing Mai 16	1/7+8/2+12
C454	Xu Mai 6	1/7+9/2+12
C455	Zhou Mai 26	1/7+9/5+10
C456	Ke 9328	N/7+8/5+10
C457	Quan Mai 4	1/7+9/2+12
C458	Quan Mai 523	1/7+9/2+12
C459	Ji Mai 181	N/7+8/5+10
C460	Zhong Mai 817	N/7+8/2+12
C461	Ji Mai 817	N/7+8/5+12
C462	Pu Bing 1	N/7+8/2+12
C463	Jing Hua 93	N/7+9/2+12
C464	Xi Nong 126	1/7+8/2+12
C465	Pu Bing 2	N/7+8/2+12
C466	Rong You 5386	1/13+16/5+10
C467	Heng Guan 35	N/7+9/2+12
C468	Zhong Mai 145	N/7+8/2+10
C469	Fu Mai 1576	1/7+9/2+12
C470	Heng 5-29	N/7+9/2+12
C471	Ai 128	N/7+8/5+10
C472	Xing Mai 19	1/7+9/2+10
C473	Zhou Mai 27	N/7+9/5+12
C474	Huai Chuan 918	N/7+9/5+12
C475	Zheng Mai 1354	1/7+9/5+10
C476	Ji Mai 325	N/7+9/5+10
C477	Ji Mai 419	1/7+9/5+10
C478	Xi Nong 07K1321	N/7+8/2+10

C479	Zhou Mai 25	1/7+9/2+12
C480	Xi Nong 611	N/7+8/2+10
C481	Xian Mai 8	N/7+8/2+10
C482	Shi Xin 5071	N/7+8/2+12
C483	Xing Mai 19	1/7+9/5+10
C484	Zhou Mai 30	1/7+9/2+12
C485	Qiu Le 1302	N/7+8/2+12
C486	Jing Fu Mai 1	N/7+9/5+10
C487	Heng 4444	1/14+15/2+12
C488	Ke 9301	N/7+8/5+10
C489	Zhou Mai 28	1/7+9/5+12
C490	Zhong Mai 875	1/7+9/2+12
C491	Ji Mai 120	N/7+9/5+10
C492	Luo 6010	1/7+8/5+10
C493	Heng 136	N/7+9/2+12
C494	Zhou Mai 8425	1/7/2+12
C495	Shi Xin 6171	N/7+8/2+12
C496	Zhou Mai 32	1/7+9/5+10
C497	Wei Long 169	1/7+8/5+10
C498	Ji Mai 631	1/7+8/5+10
C499	Luo He 6010	N/7+8/5+10
C500	Fu Mai 128	N/7+8/2+12
C501	Lun Xuan Yi	1/7+9/2+12
C502	Xi Nong 889	N/7+9/2+12
C503	Ji Mai 22	1/7+9/2+12
C504	Heng 6632	N/7+8/2+10
131 commercial varieties from MLYWWR		
C505	Bai Nong 418	1/7+8/5+10
C506	Bai Nong 3217	1/7+8/2+12
C507	Zhu Mai 6	1/7+9/5+10
C508	Wan Mai 716	N/7+8/2+12
C509	Xian Mai 10	N/7+8/2+12
C510	Bai Nong 160	1/7+9/5+10
C511	Wan Mai 16	N/7+8/2+12
C512	Wan Mai 20	1/7+8/5+10
C513	Zheng Mai 9023	1/7+9/5+10
C514	Zheng Mai 9023-1	1/7+8/2+12
C515	Bai Nong 228	N/7+9/2+12
C516	Bai Nong Chun Guang	N/7+9/5+10
C517	Zhu Mai 669	1/7+9/2+12
C518	Zhu Mai 358	1/7+9/5+10
C519	Wan 1643	N/7+8/4+12
C520	Bai Nong 5822	1/7+9/5+10
C521	Zhu Mai 305	2*/7+9/2+12
C522	Wan Mai 19	1/7+9/2+12
C523	Xin Mai 79	1/7+8/2+12
C524	D9401	N/14+15/2+12

C525	E 2078	N/7+8/5+10
C526	E 402	N/14+15/2+12
C527	E 48359	N/7+8/2+12
C528	E 55072	N/7/5+10
C529	E 76331	N/7/2+12
C530	E 82026	1/7+8/2+12
C531	E 91-1032	N/7+8/2+12
C532	E 94-5036	N/7+9/2+12
C533	E En 1	N/7+8/2+12
C534	E En 5	N/7+8/2+12
C535	E Mai 6	N/7+8/2+12
C536	E Mai 7	N/7+8/2+12
C537	E Mai 11	1/14+15/2+12
C538	E Mai 12	N/7+8/2+12
C539	E Mai 14	1/14+15/2+12
C540	E Mai 17	N/7+9/5+10
C541	E Mai 18	1/14+15/2+12
C542	E Mai 19	N/7+9/2+12
C543	E Mai 20	N/7+8/5+10
C544	Gang 9622	1/7+8/2+12
C545	Gang 9865	N/7+9/2+12
C546	Hua 9914	1/7+8/2+12
C547	Hua Mai 8	N/7+8/2+12
C548	Jing 35	N/7+9/5+10
C549	Jing 66	1/7+8/2+12
C550	S048	N/7+9/2+12
C551	Sha Yang 9302-12	N/7+8/2+12
C552	Wan 369	N/7+9/5+10
C553	Wu Da 1032	N/7+8/5+12
C554	Wu Da 311	N/7+8/2+12
C555	Wu Da 312	1/7+8/2+12
C556	Xiang Mai 8	1/7+8/2+12
C557	Xiang Nong 46619	1/7+8/5+10
C558	Yi 95218	N/7+8/5+10
C559	Yi Bin 1	1/14+15/2+12
C560	Zheng 98	1/7+8/5+10
C561	5390	1/7/2+12
C562	13397	2*/7+8/5+10
C563	323	N/7+8/5+10
C564	8207	N/7+9/5+10
C565	86004	1/7+9/2+12
C566	97-597	N/7+8/2+12
C567	98005	N/7+8/2+12
C568	9856	N/7+8/2+12
C569	Yang 06G5	N/7+9/2+12
C570	Yang 06G86	1/7+8/2+12
C571	Yang 06-164	N/7+9/2+12

C572	Yang Fu Mai 5242-1	N/7+8/2+12
C573	Yang Fu Mai 5242-2	N/7+9/2+12
C574	Nan Nong 06y86	N/7+8/5+10
C575	Zhen 05185	1/7+9/5+10
C576	Yang Mai 18	1/7+8/2+12
C577	Hua Mai 0460-1	N/7+8/2+12
C578	Hua Mai 0460-2	N/7+9/2+12
C579	Hua Mai 0480-1	N/7+8/2+12
C580	Hua Mai 0480-2	N/7+9/2+12
C581	Ning 13	1/7+8/2+12
C582	Ning 6E125	1/7+8/2+12
C583	Ning 0569	1/7+8/2+12
C584	Ning 15-1	1/7+9/5+10
C585	Ning 15-2	1/7+8/5+10
C586	Hua Mai 2566-1	N/7+8/2+12
C587	Hua Mai 2566-2	1/7+9/2+12
C588	Hua Mai 2668	1/7+8/2+12
C589	Xiang Mai 55-1	N/7+8/2+12
C590	Xiang Mai 55-2	N/7+8/5+10
C591	Mian Mai 41-1	N/7+8/2+12
C592	Mian Mai 41-2	N/7+9/2+12
C593	Mian Mai 41-3	N/7+9/5+10
C594	Chuan Mai 50-1	N/7+9/5+10
C595	Chuan Mai 50-2	1/7+9/5+10
C596	Yang Mai 158	N/7+8/2+12
C597	Xi Fu 9	1/17+18/2+12
C598	Mian Yang 28	N/7+9/2+12
C599	Yi Bin 6	N/7+8/2+12
C600	Mian Nong 2	1/17+18/5+10
C601	Ai Mai 58	N/7+8/2+12
C602	Xu Zhou 211-1	N/7+8/2+12
C603	Xu Zhou 211-2	N/7+8/5+10
C604	Su 8637-1	1/20/5+10
C605	Su 8637-2	1/20/5+10
C606	Yang 9856-1	1/7+8/5+10
C607	Yang 9856-2	N/7+9/5+10
C608	Huai Mai 12-1	N/7+8/2+12
C609	Ning 9415	2*/7+8/5+10
C610	Ning 9231-1	N/7+9/5+10
C611	Ning 9231-2	N/7+8/2.2+12
C612	Ning 93-12-1	N/7+8/5+10
C613	Ning 93-12-2	N/7+8/5+10
C614	Ning 93-12-3	N/14+15/2+12
C615	Ning 93-12-4	1/7+8/2+12
C616	Yang 87-158	N/7+8/2+12
C617	E Mai 398	N/7+8/2+12
C618	Xiang Mai 62	1/7+9/2+12

C619	E Mai 398	N/7+8/2+12
C620	E Mai 195	2*/7+8/5+10
C621	Zhong Mai 817	N/7+8/5+10
C622	Yang Fu 4	1/7+8/2+12
C623	Mian Mai 618	N/7+9/5+10
C624	Yang Fu 5	N/7+8/2+12
C625	E Mai 18	1/20/5+10
C626	Wan You 2	1/7+8/5+10
C627	Xian Mai 8	N/7+8/2+10
C628	E Mai 195	N/7+8/5+10
C629	Yang Mai 25	1/7+8/2+12
C630	E Mai 98	1/7+8/2+12
C631	Mian Mai 07-131	N/7+9/2+10
C632	Xiang Mai 031	N/7+9/5+10
C633	Nan Nong 14-8106	N/7+8/2+12
C634	Chu 0701	N/7+8/2+10
C635	Jin Wan 999	N/7+8/2+12
133 commercial varieties from SWWR		
C636	Ke Cheng Mai 1	N/7+8/5+10
C637	Mian Mai 367	N/13+16/2+12
C638	Mian Mai 37	N/7+9/5+10
C639	Mian Mai 38	N/7+8/5+10
C640	Mian Nong 4	1/17+18/5+10
C641	Mian Yang 11	1/7+8/5+10
C642	Mian Yang 21	1/7+8/5+10
C643	Mian Yang 24	N/7+9/2+12
C644	Mian Yang 25	N/7+8/5+10
C645	Mian Yang 26	N/7+9/5+10
C646	Mian Yang 27	N/7+9/2+12
C647	Mian Yang 29	N/7+9/2+12
C648	Mian Yang 30	N/7+9/2+12
C649	Mian Yang 32	1/7+8/5+10
C650	Mian Yang 33	N/7+8/5+10
C651	Mian Yang 35	N/17+18/5+10
C652	Nei Mai 11	N/7+9/2+12
C653	Xi Ke Mai 1	N/17+18/2+12
C654	Xi Ke Mai 3	N/7+8/2+12
C655	Xi Ke Mai 5	N/7+8/2+12
C656	Ya An Zao	1/7+8/2+12
C657	Yi Mai Yi	1/7+8/5+10
C658	Yu 03062	N/7+8/5+10
C659	Yu Mai 10	N/7+8/2+12
C660	Yu Mai 11	N/17+18/5+10
C661	Yu Mai 7	1/14+15/2+12
C662	Yu Mai 9	1/14+15/5+10
C663	MR11-12	N/7+9/5+10
C664	An Mai 7	N/7+9/2+12

C665	Bi Mai 18	1/7+9/2+12
C666	Chang Mai 26	1/14+15/2+12
C667	Chang Mai 28	1/13+16/2+12
C668	Chuan Mai 30	1/7+8/2+12
C669	Chuan Mai 32	1/13+16/2+12
C670	Chuan Mai 37	N/7+9/2+12
C671	Chuan Mai 38	N/6+8/2+12
C672	Chuan Mai 42	1/6+8/5+10
C673	Chuan Mai 44	1/7+8/5+10
C674	Chuan Mai 45	N/7+9/2+12
C675	Chuan Mai 47	N/13+16/2+12
C676	Chuan Mai 51	1/7+8/2+12
C677	Chuan Mai 53	1/7+8/2+12
C678	Chuan Mai 55	N/13+16/5+10
C679	Chuan Mai 56	1/13+16/5+10
C680	Chuan Mai 58	1/6+8/2+12
C681	Chuan Mai 61	N/7+8/2+12
C682	Chuan Mai 63	1/7+8/2+12
C683	Chuan Mai 65	1/7+8/5+10
C684	Chuan Mai 67	N/6+8/2+12
C685	Chuan Mai 80	1/6+8/2+12
C686	Chuan Mai 81	1/6+8/2+12
C687	Chuan Mai 91	1/6+8/2+12
C688	Chuan Mai 92	1/6+8/5+10
C689	Chuan Nong 17	1/7+9/5+10
C690	Chuan Nong 18	N/7+9/2+12
C691	Chuan Nong 19	N/7+8/2+12
C692	Chuan Nong 21	1/6+8/5+10
C693	Chuan Nong 24	1/7+9/2+12
C694	Chuan Nong 26	N/7+8/2+12
C695	Chuan Nong 27	N/7+8/2+12
C696	Chuan Shuang Mai 1	N/7/5+10
C697	Chuan Yu 16	1/14+15/5+10
C698	Chuan Yu 17	N/7+9/2+12
C699	Chuan Yu 18	1/7/2+12
C700	Chuan Yu 19	N/7+9/2+12
C701	Chuan Yu 20	1/14+15/5+10
C702	Chuan Yu 21	1/14+15/2+12
C703	Chuan Yu 23	1/7+9/5+10
C704	Fan 6	N/14+15/2+12
C705	Gui Nong 10	N/7+9/2+12
C706	Gui Nong 28	N/7+8/5+10
C707	Guo Mai 15	N/7+8/5+10
C708	Ke Cheng Mai 2	N/14+15/2+12
C709	Ke Cheng Mai 4	N/14+15/2+12
C710	Ke Cheng Mai 5	N/7+9/2+12
C711	Le Mai 3	N/7+8/2+12

C712	Liang Mai 2	1/14+15/5+10
C713	Liang Mai 3	1/6+8/2+12
C714	Liang Mai 4	N/7+8/2+12
C715	Mian Mai 1403	N/7+8/5+10
C716	Mian Mai 228	1/6+8/2+12
C717	Mian Mai 285	N/6+8/5+10
C718	Mian Mai 39	1/17+18/5+10
C719	Mian Mai 40	N/7+9/5+10
C720	Mian Mai 41	N/7+8/5+10
C721	Mian Mai 42	N/7+9/2+12
C722	Mian Mai 43	N/7+8/5+10
C723	Mian Mai 45	N/7+8/5+10
C724	Mian Mai 46	N/7+9/5+10
C725	Mian Mai 48	N/7/5+10
C726	Mian Mai 51	N/6+8/5+10
C727	Mian Nong 7	N/7+9/2+12
C728	Mian Yang 12	N/17+18/2+12
C729	Nan 30-10	N/7+9/2+12
C730	Nan Mai 618	1/14+15/5+10
C731	Nei Mai 8	N/7+9/5+10
C732	Nei Mai 9	N/7+9/5+10
C733	Qian Mai 19	1/14+15/2+12
C734	Rong Mai 3	N/17+18/5+10
C735	Rong Mai 4	N/14+15/2+12
C736	Shu Mai 482	1/7+9/5+10
C737	Te Yan Mai Nan 88	N/14+15/5+10
C738	Xi Chang 19	1/14+15/2+12
C739	Xi Fu 12	N/17+18/5+10
C740	Xi Fu 13	N/17+18/5+10
C741	Xi Ke Mai 2	1/14+15/2+12
C742	Xi Ke Mai 4	N/7+8/2+12
C743	Xi Ke Mai 6	1/7+9/5+10
C744	Xi Ke Mai 9	N/7+9/5+10
C745	Xian Mai 99	N/7+8/5+10
C746	Zhong Ke Mai 138	1/6+8/2+12
C747	Zi Mai 1	1/14+15/2+12
C748	PZ15-21	1/14+15/2+12
C749	PZ15-3	1/7+9/2+12
C750	PZ15-4	1/14+15/2+12
C751	Chuan Mai 36	2*/7+9/5+10
C752	Chuan Mai 41	N/7+8/2+12
C753	Chuan Mai 66	N/6+8/2+12
C754	Chuan Mai 68	1/6+8/5+10
C755	Gui Nong 29	N/7+9/2+12
C756	Qian Mai 20	N/7+8/5+10
C757	Qian Xing Mai 1	1/7+9/2+12
C758	Rong Mai 2	1/14+15/2+12

C759	Yu Mai 1	1/7+8/2+12
C760	Chuan Mai 33	N/7+9/2+12
C761	Chuan Mai 46	1/7+9/5+10
C762	Chuan Mai 64	1/6+8/5+10
C763	Qian Mai 17	1/7+9/2+12
C764	Wu Yi Mai	1/7+9/2+12
C765	Yi Mai 8	1/6+8/5+10
C766	Ben Di Mai	N/7+8/2+12
C767	Mian Mai 31	1/7+8/5+10
C768	Yun Mai 34	N/7+9/2+12
158 commercial varieties from NSWR		
C769	Y20	2*/7+8/5+10
C770	Xin Chun 6	N/7+9/2+12
C771	Xin Chun 8	N/7+8/2+12
C772	Xin Chun 9	N/7+9/5+10
C773	Xin Chun 10	N/7+8/2+12
C774	Xin Chun 11	N/7+8/2+12
C775	Xin Chun 12	2*/6+8/2+10
C776	Xin Chun 13	N/13+16/2+12
C777	Xin Chun 14	N/7/2+12
C778	Xin Chun 15	1/7+8/2+12
C779	Xin Chun 16	N/7+8/2+12
C780	Xin Chun 17	N/7+9/5+10
C781	Xin Chun 18	N/7+8/2+12
C782	Xin Chun 19	N/7+8/5+10
C783	Xin Chun 20	N/7+8/2+12
C784	Xin Chun 21	N/7+8/2+12
C785	Xin Chun 24	1/17+18/2+12
C786	Xin Chun 25	N/17+18/2+12
C787	Ning Chun 6	1/17+18/5+10
C788	Ba Chun 6	N/7+8/2+12
C789	Chang Chun 6	1/7+8/2+12
C790	Tu Chun 9	1/7+8/2+12
C791	Shi Dong 7	N/7+8/2+12
C792	Shi Dong 8	N/7+8/2+12
C793	Shi Dong 9	N/6+8/2+12
C794	Xiao yan 22-1	N/7+9/2+12
C795	Xiao yan 54	1/14+15/2+12
C796	Xin Dong 17	N/7+8/2+12
C797	Xin Dong 18	N/7+8/5+10
C798	Xin Dong 22	2*/7+8/5+10
C799	Xin Dong 23	N/7+8/2+12
C800	Xin Dong 24	2*/7+8/5+10
C801	Xin Dong 24	N/7+9/5+10
C802	Xi Nong 88	1/7+8/2+12
C803	Ka Shi Bai Pi	N/7+8/2+12
C804	Xiao Hei Mai	N/7+9/5+10

C805	Long Mai 26	2*/7+9/5+10
C806	Gan Mai 32	N/7+8/2+11
C807	Gan Chun 18	N/7+8/2+11
C808	Yong Liang 12	N/7+8/2+11
C809	Long Chun 1	N/7+8/2+11
C810	Long Chun 7	N/7+8/2+11
C811	Wu Chun 1	N/7+8/2+12
C812	Gan Mai 15	N/7+8/2+12
C813	Gan Mai 23	N/7+8/2+12
C814	Gan Mai 39	N/7+8/2+12
C815	Zhang Chun 9	N/7+8/2+12
C816	Zhang Chun 13	N/7+8/2+12
C817	Zhang Chun 15	N/7+8/2+12
C818	Zhang Chun 18	N/7+8/2+12
C819	Gao Yuan 506	N/7+8/2+12
C820	A Bo	N/7+8/2+12
C821	Hui Se A Fu	N/7+8/2+12
C822	C8145	N/7+8/2+12
C823	Long Chun 10	N/7+9/2+10
C824	Gao Yuan 338	N/7+9/2+12
C825	Gao Yuan 602	N/7/2+11
C826	Zhang Chun 16	N/7/5+10
C827	Ao Li Sen Ke 14	N/22/2+12
C828	Wu Chun 2	1/7+8/5+10
C829	Gan Chun 20	1/7+8/5+10
C830	Gan Mai 42	1/7+8/5+10
C831	1032 Zhuang Feng	1/7+8/5+10
C832	Gan Mai 60	1/7+8/2+11
C833	Jin 214	1/7+8/2+11
C834	Gan Mai 8	1/7+8/2+12
C835	Long Chun 8	1/7+8/2+12
C836	Wu Chun Hong	1/7+8/2+12
C837	Zhang Chun 10	1/7+8/2+12
C838	A Fu	1/17+18/2+11
C839	Yong Liang 4	1/17+18/5+10
C840	Long Mai 27	2*/7+9/5+11
C841	Gan Mai 33	N/7+8/2+11
C842	Gan Chun 19	N/7+8/2+11
C843	Yong Liang 13	N/7+8/2+11
C844	Long Chun 13	N/7+8/2+11
C845	8133	1/7+9/2+12
C846	879	1/7+8/2+12
C847	0906－19	N/7+9/2+12
C848	938	N/7+9/2+12
C849	C14	1/7+9/2+12
C850	E40－2	1/7+8/2+12
C851	E4－8	1/7+8/2+12

C852	E80－1	1/7+8/2+12
C853	E81－12	1/7+8/2+12
C854	E85－2	N/7+9/2+12
C855	F108－11	1/7+8/2+12
C856	F2－6	N/7+9/2+12
C857	F79	1/7+8/2+12
C858	G102－2	1/7+8/2+12
C859	G16	N/7+8/2+12
C860	G32－2	1/7+8/2+12
C861	G35－9	1/7+9/2+12
C862	G55－6	1/7+8/2+12
C863	G95－2	1/7+8/2+12
C864	G95－4	1/7+8/2+12
C865	Guan 136	N/7+9/5+10
C866	Kang Bing R138	1/7+8/5+10
C867	Lan Hang 122	N/7+8/2+12
C868	Lan Tian 01-368	1/7+9/2+12
C869	Lan Tian 722	1/7+8/2+12
C870	Lin You 8161	1/7+9/2+12
C871	Ling Xuan 6	1/7+8/2+12
C872	Long Jian 101	1/7+8/5+10
C873	Long Jian 107	N/7+9/2+12
C874	Long Jian 108	1/7+8/2+12
C875	Long Jian 110	1/7+8/2+12
C876	Long Jian 111	1/7+8/5+10
C877	Long Jian 112	1/7+8/5+10
C878	Long Jian 113	N/7+8/2+12
C879	Long Jian 114	1/7+8/2+12
C880	Long Jian 115	1/7+8/5+10
C881	Long Jian 116	1/7+8/5+10
C882	Long Jian 117	1/6+8/5+10
C883	Long Mai 079	1/7+9/2+12
C884	Long Mai 479	1/7+8/2+12
C885	Long Mai 491	N/20X+20Y/N
C886	Long Mai 844	1/7+9/2+12
C887	Long Mai 847	1/7+9/2+12
C888	Long Yu 0825	1/7+8/2+12
C889	Long Yu 4	N/7+9/2+12
C890	Long Zhong 6	1/7+8/2+12
C891	Long Zi Mai 1	1/7+8/2+12
C892	Long Zi Mai 2	1/7+8/2+12
C893	Xi Ping 1	1/7+8/2+12
C894	3249	N/7+9/2+12
C895	Gao You 8901	1/7+9/5+10
C896	Ka Shan 14	N/7+9/5+10
C897	Ka Shan 15	1/7+8/5+10
C898	Ka Shan 16	1/7+9/5+10

C899	Ka Shan 16210	2*/7+8/5+10
C900	Ka Shan 23	1/7+9/5+10
C901	Ka Shan 24	N/7+9/2+12
C902	Ka Shan 25	N/7+9/5+10
C903	Ka Shan 26	N/7+9/2+12
C904	Ka Shan 27	N/7+9/2+12
C905	Ka Shan 28	1/7+8/5+10
C906	Ka Shan 29	2*/7+8/5+10
C907	Ke Yi 12-6105	1/7+9/5+10
C908	Lan Kao Ai Te Zao	1/13+16/2+12
C909	Ning Mai 12	N/7+9/2+12
C910	Pu Bing 151	1/7+9/2+12
C911	Pu Bing 322	1/7+8/2+12
C912	Pu Bing 9946	1/7+8/5+10
C913	Tai Shan 9819	N/7+8/5+10
C914	Wu 44	1/7+8/5+10
C915	Wu 51	1/7+9/5+10
C916	Jing Hong 10	2*/17+18/5+10
C917	Xin Chun 7-1	N/7+8/5+10
C918	Xin Chun 7-2	2*/7+8/5+10
C919	Gan Chun 18	N/7+8/2+12
C920	Gan Chun 11	1/7+8/2+12
C921	Shi Dong 3	N/6+8/2+12
C922	Zhong Zhi 1	1/7+8/5+10
C923	Zhong Zhi 3	1/7+8/2+12
C924	Zhong Zhi 5	1/7+9/2+12
C925	Zhong Zhi 6	N/7+9/2+12
C926	Long Fu 2	N/6+8/5+10

HWWR: Huang-huai winter wheat region,

MLYWWR: Middle and Low Yangtze Winter Wheat Region,

SWWR: Southwest Winter Wheat Region,

NSWR: Northwest Spring Wheat Region.
